# Transcriptional control by two leucine-responsive regulatory proteins in *Halobacterium salinarum *R1

**DOI:** 10.1186/1471-2199-11-40

**Published:** 2010-05-28

**Authors:** Rita Schwaiger, Christoph Schwarz, Katarina Furtwängler, Valery Tarasov, Andy Wende, Dieter Oesterhelt

**Affiliations:** 1 Max Planck Institute of Biochemistry, Department of Membrane Biochemistry, Am Klopferspitz 18, 82152 Martinsried, Germany; 2 School of Life Sciences, Arizona State University, Tempe, Arizona 85287, USA; 3 QIAGEN GmbH, Qiagen Strasse 1, 40724 Hilden, Germany

## Abstract

**Background:**

Archaea combine bacterial-as well as eukaryotic-like features to regulate cellular processes. *Halobacterium salinarum *R1 encodes eight leucine-responsive regulatory protein (Lrp)-homologues. The function of two of them, *Irp *(OE3923F) and *lrpA1 *(OE2621R), were analyzed by gene deletion and overexpression, including genome scale impacts using microarrays.

**Results:**

It was shown that Lrp affects the transcription of multiple target genes, including those encoding enzymes involved in amino acid synthesis, central metabolism, transport processes and other regulators of transcription. In contrast, LrpA1 regulates transcription in a more specific manner. The *aspB3 *gene, coding for an aspartate transaminase, was repressed by LrpA1 in the presence of L-aspartate. Analytical DNA-affinity chromatography was adapted to high salt, and demonstrated binding of LrpA1 to its own promoter, as well as L-aspartate dependent binding to the *aspB3 *promoter.

**Conclusion:**

The gene expression profiles of two archaeal Lrp-homologues report in detail their role in *H. salinarum *R1. LrpA1 and Lrp show similar functions to those already described in bacteria, but in addition they play a key role in regulatory networks, such as controlling the transcription of other regulators. In a more detailed analysis ligand dependent binding of LrpA1 was demonstrated to its target gene *aspB3*.

## Background

The basal transcription apparatus in Archaea shows similarity to the eukaryotic RNA polymerase (RNAP) II system [1-4]. Archaeal promoter sequences and the core proteins RNA polymerase (RNAP), TATA-binding protein (TBP), and the transcription factor IIB homologue (TFB) are structurally and functionally related to their eukaryotic counterparts [2,5]. Although the basal transcriptional complex is composed of eukaryotic-like components, archaeal regulatory proteins are often homologous to bacterial regulators [6]. One group of bacterial regulators which have been found in all archaeal genomes belongs to the Lrp/AsnC family (leucine-responsive regulatory protein (Lrp), asparagine synthase C (AsnC)). *Escherichia coli *Lrp is the most extensively studied member in bacteria [7,8] and controls the expression of up to 75 target genes. As a global regulator of transcription, Lrp is believed to coordinate cellular metabolism in response to nutritional and environmental alterations [9]. Most of these genes are involved in amino acid metabolism. Lrp can bind to DNA in its homodimeric form and either represses or activates transcription, modulated by the effector molecule L-leucine. Negative autoregulation of Lrp, however, occurs in a leucine independent way [10].

Genes encoding putative Lrp/AsnC-homologues have been studied in several archaea [11], including *Methanocaldococcus jannaschii *[12-14], *Sulfolobus *species [15-17] and *Pyrococcus *species [18,19]. As demonstrated for the *Sulfolobus solfataricus *Ss-Lrp and Lrs14, those Lrp/AsnC-homologues were shown to bind to their own promoter regions, thereby repressing transcription [20-22]. Besides controlling its own gene expression, *Sulfolobus solfataricus *Ss-LrpB positively regulates the pyruvate ferredoxin oxidoreductase (POR) encoding operon and two permease genes [23,24]. Another Lrp-like protein, LysM from *S. solfataricus*, regulates the expression of the *lysWXJK *operon encoding lysine biosynthetic enzymes. In fact, *in vitro *binding of LysM to the *lysW *promoter takes place only if lysine is absent [25]. Footprint analysis of the Sa-Lrp gene from *Sulfolobus acidocaldarius *revealed multiple binding sites in the promoter region [26], a pattern that had been described earlier for bacterial Lrp proteins [27]. Leucine has been suggested as a possible cofactor for Sa-Lrp under certain physiological conditions [26].

In *M. jannaschii*, not only do the Lrp-like proteins Ptrl and Ptr2 regulate their own transcription, but Ptr2 can activate transcription of the ferredoxin (*fdx*) and the rubredoxin (*rb2*) genes by facilitating recruitment of TBP to their promoters [13]. In a *Pyrococcus furiosus *cell-free transcription system, LrpA exerts negative autoregulation of its own transcription [19] by interfering with the recruitment of RNA polymerase [28]. Crystal structures determined for several bacterial and archaeal Lrp-like proteins (for an overview, see [29] show that they contain a N-terminal helix-turn-helix DNA-binding domain (HTH). A flexible hinge connects this domain with the C-terminal oligomerization and effector binding domain [30-32]. The latter forms a so-called RAM-domain (regulation of amino acid metabolism-domain) [33], designed to bind an effector molecule in the interface between the two dimers. A structure alignment of archaeal and bacterial Lrp-homologues is shown in additional file 1.

In halophilic archaea relatively little is known about Lrp-like-regulators. The current study focuses on the Lrp-like-regulators, LrpA1 and Lrp in *H. salinarum *R1. To identify Lrp-targets, deletion mutants (Δ*lrp*, Δ*lrpA1*) as well as strains upregulated in these genes (↑*lrp*, ↑*lrpA1*) were compared pairwise against the parental strain R1 by DNA-microarrays. These results demonstrated that Lrp exerts a global transcriptional control in this organism. On the other hand, LrpA1 was shown to possess a specific regulatory function targeting the aspartate transaminase gene (*aspB3*). We demonstrated effector molecule dependent binding of LrpA1 to the *aspB3 *promoter by DNA-affinity chromatography, as well as effector molecule dependent gene expression of *aspB3 *by northern analysis.

## Results

### Specific transcriptional control by LrpAl in *H. salinarum *R1

Of the eight Lrp-homologues found *H. salinarum, lrpA1 *and *lrp *are located next to genes involved in amino acid metabolism (*aspB3 *aspartate transaminase, *glnA *glutamine synthetase), suggestive of a direct regulatory influence. To confirm this, and to identify other possible targets of LrpA1 we used DNA-microarrays. Two genetic approaches, either a deletion strain of *lrpA1 *(Δ*lrpA1*) or an overexpressing strain (↑*lrp*A1), were compared pairwise against the *H. salinarum *R1 parental strain. Target genes showing reciprocal regulatory changes between the deletion strain and the overexpression strain, reflect the regulatory effects of LrpA1 (Table 1). The deletion and overexpressing strains grew as well as the parental strain in complex medium (additional file 2). The deletion mutant was verified by southern blot analysis (additional file 3). Induction of *lrpA1 *transcription in the overexpressing strain was shown by microarray-analysis. Level of lrpA1 expression was 24-fold higher in overexpression mutant than in wild type (Table 1) and a complete list of significantly differentially expressed genes is presented in (additional file 4)

**Table 1:** Differentially expressed genes in Δ*lrpA1 *and ↑*lrpA1*

	Δ*lrpA1*	↑*lrpA1*		
ID	-fold change	-fold change	gene	name
*Transcriptional regulators (REG)*				
OE2621R	-3.2	24.4	*lrpA1*	transcription regulator LrpA1

*Amino acid metabolism (AA)*				
OE2619F	5.1	-1.2	*aspB3*	aspartate transaminase

*Transcription (TC)*				
OE2084R	1.4	-2.4	*tfbB*	transcription initiation factor TFB

*Miscellaneous (MIS)*				
OE6130F	6.7	-6.3	-	conserved hypothetical protein

As expected, transcription of *aspB3*, the gene adjacent to *lrpA1*, was affected by the absence or overexpression of *lrpA1*. In the ↑*lrpA1 *background (Table 1), *aspB3 *showed slight repression, while deletion of *lrpA1 *led to strong induction. Another LrpA1 target gene identified by the microarray analysis was *tfbB*, the basal transcriptional regulator gene. This gene was repressed by LrpA1-overexpression. Additionally, strong repression was found for OE6130F, the gene encoding a hypothetical protein of unknown function. OE6130F is located on the plasmid PHS2. Its adjacent genes are OE6128R encoding a conserved hypothetical protein and OE6133R encoding a transposase.

In the Δ*lrpA1 *mutant, induction of *aspB3 *and OE6130F was confirmed by RT-qPCR, which showed reductions in transcript levels of 19- and 28-fold, respectively (additional file 5)

### Organisation of *lrpA1 *and *aspB3 *operons of *H. salinarum *Rl

**Figure 1:**
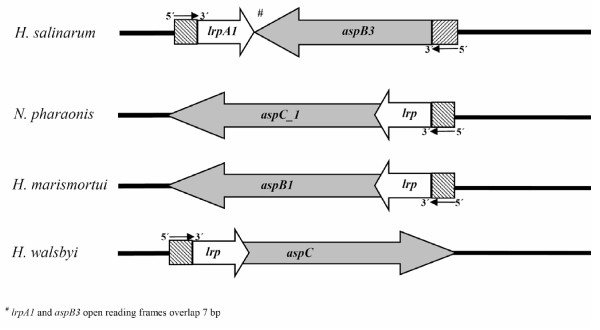
**Arrangement of *lrp *and *asp genes *in halophilic organisms**. In *H. salinarum lrpA1 *and *aspB3 *have separate promoters and the genes are orientated in opposite directions. *lrp *and *asp *in *N. pharaonis, H. marismortui *and *H. walsbyi *have one promoter and their open reading frames overlap.

Since the DNA-microarray analysis showed that *aspB3 *is the most prominent target for LrpA1, we performed further investigations on the regulation of *aspB3 *by LrpA1. *LrpA1 *and *aspB3 *are orientated in opposite directions and have separate promoters. In other halophilic organisms, like *Natronomonas pharaonis*, *Haloquadratum walsbyi *and *Haloarcula marismortui*, these genes are orientated in the same direction and share one common promoter (Fig 1).

**Figure 2:**
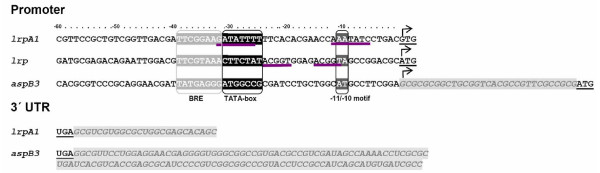
**Mapping of 5'UTR (*lrpA1, lrp, aspB3*) and 3'UTR (*lrpA1, aspB3*)**. The transcriptional start site is marked by an arrow, the start codon is underlined. Putative TATA-box, BRE-element and the -11/-10-motif are boxed. Possible DNA-binding motifs are underlined (violet). The 5'leader sequence of *aspB3 *is shaded in grey. 3'UTR of *lrpA1 *and *aspB3 *are shaded in grey, the stop codon is underlined.

The transcription start sites and 3'ends of *lrpA1 *and *aspB3*, were located using 5'3'-RACE, based on the circularisation of RNA. *lrpA1 *was found to be transcribed as a leaderless mRNA, starting at the first G of the start codon, GTG (Fig. 2). The putative TATA-box of *lrpA1 *is located -27 bp upstream of the transcriptional start site (Fig. 2). Possible regulator protein binding sites were found at positions -31 to -25 and -12 to -6. In *M. jannaschii *AT rich inverted repeat sequences have been demonstrated to be DNA-binding motifs for Lrp-like transcriptional regulators [12]. Another cis-element in the *lrpA1 *promoter is two adenines at position -11/-10, consistent with the basal promoter motif previously described [34]. As a consequence of the overlap of these genes, the *lrpA1 *3' untranslated region (3'UTR) shows complementarity to the ORF of *aspB3 *over 25 bp (Fig. 2), a consequence of the overlap of these genes. No uridine rich terminator sequence was identified for the *lrpA1 *transcript (Fig. 2).

In contrast to *lrpA1*, the *aspB3 *transcript has a 5'UTR leader sequence of 31 bp, without a Shine-Dalgarno sequence upstream of the AUG start codon (Fig. 2). Inspection upstream of the *aspB3 *transcription start site revealed, that the *aspB3 *promoter does not contain a consensus TATA-box at the expected position (-24 to -27). The 3'UTR of *aspB3 *included a 127 bp terminal sequence that is complementary to the 3'end of *lrpA1*. No characteristic termination signal was detected in this region (Fig. 2).

### LrpAl binds to the *aspB3 *and to its own promoter

**Figure 3:**
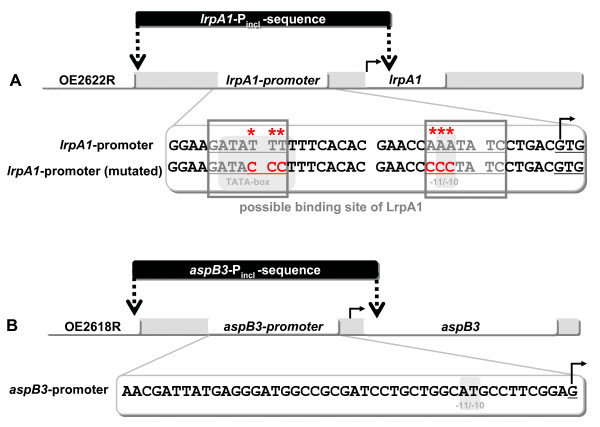
**Promoter region of *lrpA1 *and *aspB3***. *LrpA1*-P_incl _and *aspB3*-P_incl _are the DNA fragments used for Protein-DNA interaction studies, including the complete promoter region of *lrpA1 *and *aspB3*, respectively (A,B). The mapped transcription start site is marked by an arrow (A,B). The inverted repeat in the *lrpA1 *control region demonstrates a possible protein binding site for LrpA1 (grey frame). Mutations in the control region are marked by red asterisks (A). TATA-box (A) and the -11/-10-motif (A,B) are shaded in grey.

**Figure 4:**
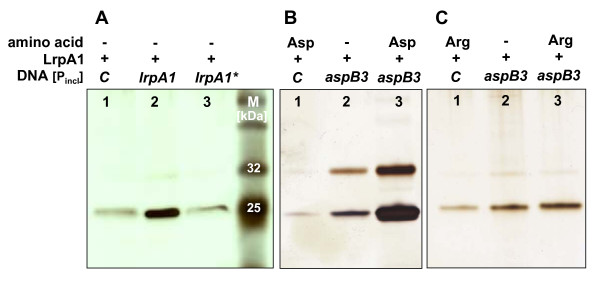
**Effects of LrpA1 binding to promoter fragments**. Analytical DNA-affinity chromatography showing binding of LrpA1 to DNA fragments (*lrpA1*-P_incl_, *aspB3*-P_incl _and control DNA *flgB1*) analyzed by SDS-PAGE. The lower band on the gel shows the monomer of LrpA1, whereas the upper band is the dimeric form. Binding of LrpA1 to non specific DNA control (*flgB1*) (lane 1), to *lrpA1*-P_incl _(lane 2) and to mutated *lrpA1*-P_incl _(lane 3), with an altered inverted repeat sequence (see Fig. 3A) (A). LrpA1 binding to non specific control DNA (*flgB1*) in the presence of 5 mM L-aspartate (lane 1), to the *aspB3*-P_incl _(lane 2) and to the *aspB3*-P_incl _in the presence of 5 mM L-aspartate (B). Binding of LrpA1 to non specific DNA (*flgB1*) with 5 mM L-arginine (lane 1) and to the *aspB3*-P_incl _without (lane 2) and with (lane 3) 5 mM L-arginine (C).

Several bacterial and archaeal Lrp-homologues are known to bind to their own promoter as well as to the promoters of target genes [10,11,35]. The binding of LrpA1 to its own promoter and to the *aspB3 *promoter region was examined using analytical DNA-affinity chromatography, adapted to halophilic conditions. Because the exact binding sites for LrpA1 in the promoter regions of *lrpA1 *and *aspB3 *were unknown, the complete non-coding region upstream of these two genes was amplified. These PCR products were designated as *lrpA1*_Pincl _and *aspB3*_Pincl_; (promoter sequence inclusive; Fig. 3A,B) with a length of 234 bp and 208 bp, respectively. An additional PCR product was generated where the inverted repeat sequence in the *lrpA1 *promoter region was mutated (indicated by red asterisk in Fig. 3A). There was no corresponding inverted repeat sequence in the *aspB3 *promoter (Fig. 3B). As a non-specific binding control in the assay, we used the flagellin gene *flgB1*. The DNA fragments *lrpA1*_Pincl_, *aspB3*_Pincl _and the control fragment *flgB1 *were amplified using a biotin labelled primer and subsequently coupled to a streptavidin sepharose matrix. Heterologously expressed LrpA1, tested for correct folding by CD-spectroscopy and size exclusion chromatography (additional file 6), was then incubated with DNA fragments and eluted protein fractions analyzed on SDS-PAGE (Fig. 4).

As shown in Fig. 4A, LrpA1 binds to the *lrpA1*_Pincl _fragment. Mutation of the inverted repeat in this sequence (indicated by asterisks in Fig. 3A) prevented binding of LrpA1 (Fig. 4A). In conjunction with the current knowledge about other Lrp-homologues, these results suggest LrpA1 is subject to negative autoregulation [11]. A weaker binding of LrpA1 to the *aspB3*_Pincl _fragment was also demonstrated (Fig. 4B). Lrp-homologues often control gene expression together with a ligand molecule. Therefore we tested aspartate as a possible effector molecule of the *aspB3 *gene expression. 5 mM aspartate was added to the binding experiment, resulting in significantly enhanced binding of LrpA1 to the *aspB3*_Pincl _sequence (Fig. 4B). If 5 mM arginine was used instead of aspartate, the binding efficiency of LrpA1 to the *aspB3*_Pincl _fragment was not enhanced (Fig. 4C), indicating that the interaction shows specificity for aspartate. Table 2 shows the relative binding efficiencies of LrpA1 to sepharose-bound DNA fragments calculated from 3 independent binding experiments. Both lanes, the monomer and the dimer, were included in our calculations of band densities. According to the estimated molecular weight, the upper band represents protein dimers. The presence of LrpA1 dimers after treatment with heat and SDS indicates that they are stable to these denaturing conditions. Thus, LrpA1-DNA binding studies showed that LrpA1 binds to its own promoter, as well as to the *aspB3 *promoter enhanced by aspartate (Fig. 4).

**Table 2:** Relative binding efficiencies of LrpA1 to sepharose-bound DNA fragments

DNA-fragment	band density	%^a^
*lrpA1*-P_incl_	846 ± 280	100%
*lrpA1*-P_incl_(mutated)	201 ± 33	24%
control-DNA (C)	313 ± 70	37%
*aspB3*-P_incl _(+ 5 mM asp)	1404 ± 228	100%
*aspB3*-P_incl_	473 ± 220	34%
control-DNA (+ 5 mM asp) (C)	92 ± 33	7%
*aspB3*-P_incl _(+5 mM arg)	175 ± 94	100%
*aspB3*-P_incl_	52 ± 22	77%
control-DNA (+ 5 mM arg) (C)	135 ± 69	30%

^a ^Average and standard deviation values are based on three separate experiments

### LrpA1 regulates transcription of *aspB3 *in an aspartate dependent manner

*H. salinarum *possesses three different aspartate transaminases, AspB1, AspB2 and AspB3 (see sequence comparison in additional file 7). All belong to subgroup Ib of the aspartate transaminases [36], and share 35, 37 and 32% sequence identity, respectively, with the *Thermus thermophilus *enzyme [37]. Aspartate transaminases catalyze the reversible conversion of aspartate and oxoglutarate to oxaloacetate and glutamate.

**Figure 5:**
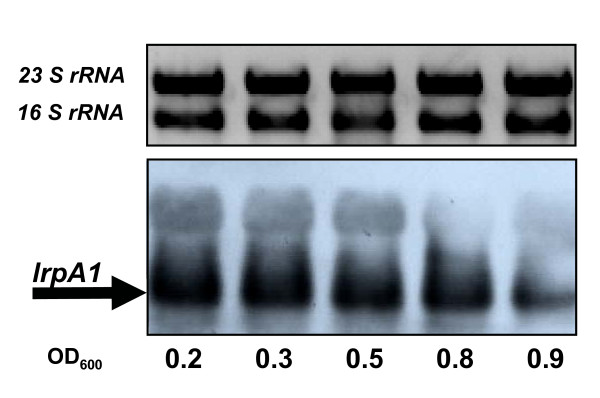
**Northern analysis of the *lrpA1 *transcript level**. The upper panel represents 16S and 23S rRNA bands on a 1% agarose gel after ethidium bromide staining. The lower panel displays detected transcripts (429 kb; marked by an arrow) using a probe against *lrpA1. H. salinarum R1 *wild type cells were grown in complex media and harvested at the indicated OD-values.

**Figure 6:**
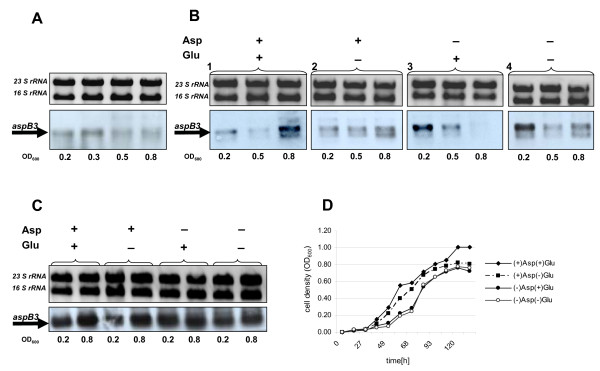
**Northern analysis of the *aspB3 *transcript level**. The upper panel represents 16S and 23S rRNA bands on a 1% agarose gel after ethidium bromide staining. The lower panel displays detected transcripts (1107 kb; marked by an arrow) using a probe against *aspB3*. *H. salinarum R1 *wild type cells were grown either in complex (A) or synthetic media (B) and harvested at the indicated OD-values. Cells of Δ*lrpA1 *grown in synthetic media were harvested at indicated OD-values (C). Synthetic media (B1-4; C) was supplemented with aspartate and/or glutamate as indicated (+/-). Growth curve of *H. salinarum R1 *grown in synthetic medium with an amino acid composition as indicated (D).

Since LrpA1 appears to regulate the expression of *aspB3*, we first investigated the transcription of *lrpA1 *during different growth phases using northern blot hybridization and a specific probe against *lrpA1 *(429 bp) (Fig. 5). In the wild type strain, *lrpA1 *transcripts remained constant at cell densities of 0.2 to 0.8 (OD_600_). Although the *lrpA1 *transcript could be detected during stationary phase, its amount decreased (Fig. 5). These results show that *lrpA1 *transcripts accumulate during exponential growth. Therefore, to test the regulation of *aspB3 *by LrpA1 in an aspartate and glutamate dependent manner, wild type and the Δ*lrpA1 *cells were cultivated up to cell densities in the range of 0.2 to 0.8 (OD_600_) either in a complex medium or in a synthetic medium, in the presence or absence of aspartate or glutamate. RNA was extracted and analyzed on northern blots using an *aspB3 *specific probe (Fig. 6A-6B). This hybridized to a corresponding transcript with the expected size of *aspB3 *(1107 nt). In complex medium, *aspB3 *was slightly induced at the beginning of the exponential growth phase (Fig. 6A). In contrast, in synthetic medium supplemented with aspartate and glutamate, *aspB3 *was induced in the early stationary phase (OD_600 _= 0.8) (Fig. 6B-1). While in synthetic medium with aspartate a slight induction of *aspB3 *was observed in the early stationary phase (Fig. 6B-2). In synthetic medium with glutamate, *aspB3 *was already abundant in the early exponential growth phase (OD_600 _= 0.2) (Fig. 6B-3). When both amino acids (asp, glu) were omitted, *aspB3 *showed high induction at a cell density of 0.2 and slight induction at 0.8 (Fig. 6B-4).

In synthetic medium, *H. salinarum *showed similar growth behaviour whether or not aspartate was present (Fig. 6D). These results show that *aspB3 *transcription is repressed in the presence of aspartate. If there is no aspartate in the synthetic medium (Fig. 6B-3,4), or it has been metabolized in the early stationary phase, repression is released, and synthesis of aspartate from glutamate ensues.

At cell densities of ≥ 10^9 ^cells/ml (OD_600 _≥ 1.0) *H. salinarum *R1 has been reported to rapidly metabolize aspartate [38]. Previously reported growth studies of *H. salinarum *R1 have shown that when media have both aspartate and glutamate present, the former amino acid is metabolized rapidly while the levels of the latter remains constant [38]. To test the regulatory effect of LrpA1 on the *aspB3 *gene transcription, mRNA levels of *aspB3 *were analyzed in Δ*lrpA1 *cells grown with or without aspartate or glutamate (Fig. 6C). We observed an increased and constitutive transcription, independent of the added amino acids. This demonstrates unambiguously the involvement of LrpA1 in the regulation of the *aspB3 *gene expression.

### Multiple transcriptional control by Lrp in *H. salinarum*

Residues in the sequence of LrpA1 and Lrp predicted to be involved in ligand specificity of the binding pocket are different in both regulators (additional file 1) and therefore a different control of targets was expected. *lrp *is located next to the glutamine synthetase gene *glnA*, without a sequence overlap. The mapped transcription site is at the A of the start codon ATG (Fig. 2). Target genes for Lrp were identified using the same approach as described for LrpA1 (additional file 2, 3). Target genes showing reciprocal regulatory changes between the deletion strain (Δ*lrp*) and the overexpression strain (↑*lrp*), suggesting the direct regulatory effects of Lrp (Table 3). Induction of *lrp *transcription in overexpressing strains was shown by microarray-analysis. In the overexpression mutant, levels of lrp is 46-fold higher than in wild type (Table 3). The successful overexpression of Lrp was proven by western blot analysis using a specific antibody against Lrp (additional file 8). A complete list of significantly differentially expressed genes is presented in additional file 9.

**Table 3:** Differentially expressed genes in Δ*lrp *and ↑*lrp*

	Δ*lrp*	↑*lrp*		
ID	-fold change	-fold change	gene	name
*Transcription (TC)*				
OE1478R	-1.6	1.7	*tfbF*	transcription initiation factor TFB

*Transport processes (TP)*				
OE1678R	-1.8	1.6	*pstC2*	ABC-phosphate transporter permease
OE3908R	-1.8	1.8	*phnC*	ABC-phosphate transporter ATP binding protein
OE4301R	-2.2	1.8	*dppF1*	ABC-peptide transport ATP binding protein
OE4302R	-2.4	1.9	*dppD1*	ABC-peptide transport ATP binding protein
OE4303R	-1.9	1.8	*dppC1*	ABC-peptide transport permease
OE4552F	-2.3	2.0	*dppB2*	ABC-peptide transport permease

*Amino acid metabolism (AA)*				
OE3922F	-1.5	2.1	*glnA*	glutamine synthetase

*Central intermediary processes (CIM)*				
OE1710R	-1.9	2.0	*korB*	oxoglutarate ferredoxin oxidoreductase β-subunit
OE1711R	-2.9	2.3	*korA*	oxoglutarate ferredoxin oxidoreductase α-subunit
OE5160F	2.5	-1.9	*gldA1*	glycerole dehydrogenase

*Signal transduction (SIG)*				
OE5243F	1.7	-2.7	*car*	transducer protein Car

**Figure 7:**
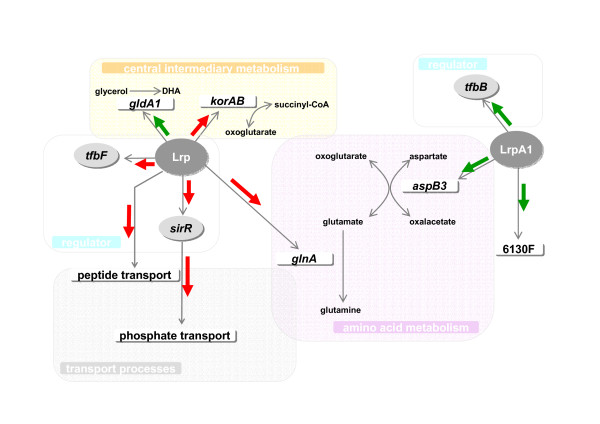
**Overview of the regulatory effects of LrpA1 and Lrp**. Metabolic pathways found to be regulated by Lrp and LrpA1, respectively, including genes encoding enzymes involved in amino acid metabolism, regulator genes and genes involved in transport processes. (red arrows mean induction and green arrows mean repression; grey arrows represent possible effects by a Lrp-homolog).

Besides genes of the amino acid metabolism, the targets affected by Lrp were genes of central intermediary metabolism, (Fig. 7; Table 3). For example, *glnA*, the gene next to *lrp*, was induced in the Lrp-overexpression strain. The glycerol dehydrogenase gene, *gldA1 *was repressed by Lrp, whereas *korAB*, encoding the oxoglutarate oxidoreductase complex, which is part of the TCA-cycle was induced. Additionally the *car *gene, encoding a transducer protein involved in signal transduction processes was repressed in the presence of Lrp-overexpression. Genes involved in transcriptional regulation were also affected by Lrp. The transcriptional regulator *sirR*, a homologue of the staphylococcal iron regulator repressor and the basal transcription factor gene *tfbF*, were found to be induced by Lrp-overexpression (Fig. 7; Table 3) [39]. Lrp-overexpression produced induction of transporter genes, like *pstC2 *and *phnC*, which belong to phosphate transport operons (Fig. 7; Table 3).

## Discussion

Lrp-homologues have been described for several bacterial and archaeal organisms, but not yet for halophilic archaea. Here we investigated the function of a halophilic Lrp-homologue, LrpA1. Additional file 1 shows an alignment of LrpA1 with other archaeal and bacterial Lrp-homologues. High sequence similarity was observed with LrpA (PF1601) from *P. furiosus*. The low sequence identity in the ligand binding pocket, named RAM-domain (additional file 1; β3β4) between LrpA1 and Lrp suggests different regulatory mechanisms (additional file 1). The *H. salinarum *LrpA1 binding pocket belongs to a subgroup of the Lrp-like proteins, which includes some that might be effector-independent [32] and some for which the effector regulation is unknown (additional file 1). The Lrp ligand binding site shows high amino acid conservation with *S. solfataricus *LysM which probably binds lysine [25] (additional file 1). We therefore expect the *H. salinarum *Lrp to be a ligand dependent regulator, which is a subject of future investigation.

**Figure 8:**
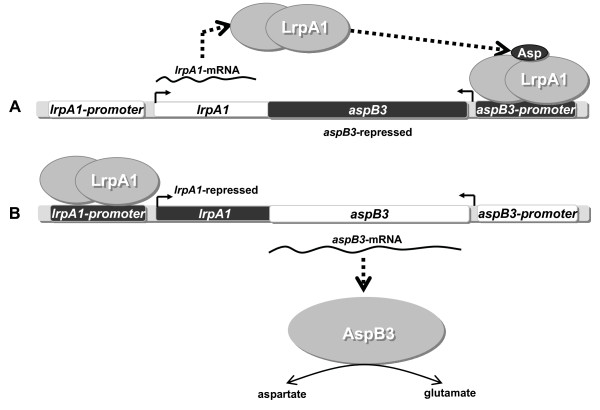
**Reciprocal regulation of the *lrpA1 *and the *aspB3 *gene expression**. LrpA1 binds together with aspartate to the *aspB3 *promoter in order to prevent *aspB3 *gene expression (A). Once aspartate is metabolized in the medium LrpA1 no longer has the ability to bind to the *aspB3 *promoter. Instead, *aspB3 *gene expression will be activated to generate aspartate from glutamate. Without aspartate LrpA1 binds to the *lrpA1 *promoter to repress *lrpA1 *gene expression (B).

LrpA1 was shown to be regulated by aspartate and since this protein is a specific regulator of two different promoter sequences, *lrpA1 *and *aspB3*, we hypothesize that the *lrpA1 *and *aspB3 *gene expression is reciprocally regulated (Fig. 8). For LrpA1, we suggest an inverted repeat in the *lrpA1 *promoter as a putative protein binding site, whereas the *aspB3 *promoter lacks such a sequence. At first glance, it seems surprising that LrpA1 binds to two different promoter structures, but as shown for *E. coli *Lrp, promoter target sites may share only weak sequence conservation [7]. In the exponential phase *lrpA1 *expression is maximal. Since L-aspartate is present in the medium the binding of LrpA1 to the *aspB3 *promoter is enhanced. Once aspartate is metabolized, small conformational changes in LrpA1 might occur that allow it to bind to the *lrpA1 *promoter (Fig. 8). As the repression of the *aspB3 *gene is abrogated, transcription of aspartate transaminase will be initiated in order to synthesize aspartate from glutamate (Fig. 8). This model of LrpA1 regulation could explain a direct influence of LrpA1 in regulating its neighbour gene *aspB3*. The DNA-microarray data indicate that LrpA1 regulates the expression of *aspB3*.

In contrast to LrpA1, another Lrp-homologue named Lrp affects the transcription of genes encoding proteins involved not only in amino acid but also in central metabolism. For many organisms, Lrp acts as both an activator and a repressor of transcription. Like *E. coli *Lrp, the Lrp of *H. salinarum *R1 affects the regulation of amino acid metabolism and genes encoding peptide transporter *dpp *(Fig. 7; Table 3). Lrp binding sites in *H. salinarum *NRC-1, a strain that shows a 99.9% sequence identity to *H. salinarum *R1 [40], have been previously reported [40]. In NRC-1 Lrp-homologues are designated as Trh and nine of them are annotated in the genome of *H. salinarum *NRC-1. *H. salinarum *R1 LrpA1 (OE2621R) and Lrp (OE3923F) are 100% identical with the *H. salinarum *NRC-1 Trh7 and Trh4, respectively, the latter one was previously analyzed [41]. Comparison of our data with the published NRC-1 data revealed that, out of all the affected genes, only three were showing the same trends in both: the glutamine synthetase gene, *glnA*, which is located adjacent to *lrp*; the glycerol dehydrogenase gene, *gldA1 *and the transducer gene, *car *(Table 3). *GldA1 *was repressed by Lrp in *H. salinarum *R1. The metabolism of glycerol is complex. It can either be converted to dihydroxyacetone (DHA) by glycerol dehydrogenase GldA1 [42], or phosphorylated by glycerol kinase to glycerol-3-phosphate. The latter can be fed into glycolysis as dihydroxyacetone phosphate (DHAP), or is converted to glycerol-1-phosphate which is used as a substrate for the production of archaeal phospholipids. However, the fate of DHA remains unclear because the corresponding kinase for the subsequent conversion of DHA to DHAP is not yet known [43]. The repression of *gldA1 *might favour glycerol phosphorylation by reducing the flow of glycerol to dihydroxyacetone (DHA). Besides the three affected genes that were common between NRC-1 and R1, there were distinct targets of Lrp in strain R1. For example, activation of *korAB*, encoding the oxoglutarate oxidoreductase complex, a TCA cycle enzyme (Fig. 7; Table 3). KorAB belongs to the family of two oxoacid:ferredoxin oxidoreductases (OR) and catalyzes the oxidative decarboxylation of oxoglutarate and is part of the following conversion together with CoA to succinyl-CoA. For *S. solfataricus *Ss-LrpB, activation of the pyruvate ferredoxin oxidoreductases *por*-operon has been reported by Peeters; 2009 [24]. The OR-enzymes act on various substrates that play key roles in amino acid metabolism [44].

In *H. salinarum *R1, *korAB *induction by Lrp suggests that KorAB catalyzes the rate-determining step of the TCA-cycle. This might influence the oxoglutarate/glutamate balance and shift carbon flow towards glutamate synthesis or degradation. In the Lrp-overexpression strain, a slight induction was observed for the glutamate dehydrogenase gene, *gdhA2*. Glutamate is incorporated into the TCA-cycle by GdhA2 and metabolized by KorAB to generate further metabolites or provide reducing equivalents. As already mentioned, Lrp regulates the synthesis of glutamine from glutamate by induction of *glnA*. In *H. salinarum*, glutamate is accumulated as a carbon storage compound and as a compatible osmolyte, and reaches concentrations of 50-100 mM [45,46]. If needed, glutamate can be converted into other metabolites, e.g. amino acids.

Both regulators, LrpA1 and Lrp, influence the expression of *tfb*'s. It has been proposed earlier that different combinations of TFBs and TBPs may act in an analogous way to bacterial sigma factors in order to control global gene expression in *H. salinarum *NRC-1 [46-49]. Lrp activates *tfbF*, whereas LrpA1 represses *tfbB*. In strain NRC-1, TfbF is thought to control either directly or indirectly the transcription of target genes [41].

The transcriptional regulator *sirR*, a homologue of the staphylococcal iron regulator repressor, was found to be induced by Lrp (Fig. 7; Table 3) [39]. SirR is described as a repressor of a putative Mn-dependent ABC-transporter in *H. salinarum *NRC-1 [50]. In R1, induction of the putative Mn-dependent ABC-transport operon (OE5144R, OE5146R, OE5147R) in a Δ*sirR *deletion strain was shown (Schwaiger, unpublished data). In the current study slight repression of the ABC-transporter gene, OE5147R was detected in the Lrp-overexpression strain, where *sirR *is induced. This is consistent with SirR acting as a repressor of the ABC-transport operon. The data also showed induction of *pstC2 *and *phnC*, which belong to phosphate and phosphonate transport operons (Fig. 7; Table 3). In NRC-1, SirR is thought to take part in the regulation of phosphate transport processes [50]. Lrp might then indirectly influence phosphate metabolism by controlling *sirR *expression.

## Conclusion

In summary, these studies on Lrp-like homologues in the halophilic branch of archaea have clearly demonstrated that they share a similar general function to their homologues in bacteria, i.e. they are transcriptional regulators that may have narrow or global regulatory actions. Lrp activates the gene expression of the glutamine synthetase gene *glnA*, influences peptide- and phosphate transport, as well as the central intermediary metabolism, and activates the expression of the transcriptional regulator *sirR*. By the control of *sirR *gene expression through Lrp correlation between amino acid metabolism and metal dependent processes could be demonstrated. In contrast to Lrp, LrpA1 regulates gene expression of fewer genes, amongst them the aspartate transaminase gene *aspB3*. LrpA1 was shown to bind to the *lrpA1 *promoter region, as well as an aspartate dependent binding to the *aspB3 *promoter region. To gain more insights into the LrpA1 and L-aspartate dependent *aspB3 *gene expression, northern blot analysis were performed, that showed an induction of the *aspB3 *transcription in the absence of L- aspartate. This occurs either in a medium lacking aspartate or after aspartate is metabolized in the stationary phase. At the same time, an induction of the *lrpA1 *gene expression was observed. This can be illustrated in a model that postulates a reciprocal regulation of the *lrpA1 *and *aspB3 *gene expression. Much remains to be understood, but the current work provides a solid foundation for further investigations of the haloarchaeal Lrp protein family and their regulatory networks.

## Methods

### Strains and growth conditions

*H. salinarum R1 *(DSM 671) and the deletion strains (Δ*lrp*, Δ*lrpA1*) were grown in either complex or synthetic medium, as described previously [51,52]. The *E. coli *strains DH5α and BL21(DE3), used for cloning and protein expression, were grown in Luria-Bertani (LB) medium, supplemented with antibiotics when necessary [53].

### Construction of deletion and overexpression mutants in *H. salinarum*

The construction of *lrpA1 *and *lrp *deletion mutants was performed according to [54]. Briefly, oligonucleotides were used to amplify the adjacent region downstream and upstream of the gene of interest (additional file 10). The obtained PCR-products were digested with *PstI*, fused by ligation, reamplified and cloned into pMKK100 [54] using *BamHI *and *XbaI *restriction sites. The *lrp*↑ and *lrpA1*↑ strains were constructed by insertion of pKF203 and pKF204 plasmids into the *lrp *and *lrpA1 *region of *H. salinarum*, respectively. The plasmids were constructed as described in additional file 11. Deletion plasmids and overexpression plasmids were introduced into *H. salinarum *by the PEG-mediated method according to [50]. Deletion mutants were generated by a two-step procedure of selecting separate single cross over events using red-blue screening as described by [54]. The correct genotype was verified by PCR and Southern blot hybridization (additional file 3). The presence of the overexpression plasmid in each transformant was determined by PCR followed by sequencing of the amplified fragments.

### Isolation of total RNA

*H. salinarum *cells were harvested at an OD_600 _of 0.2-0.8 by centrifugation for 5 min at 12000 g (4°C). The pellet was resuspended in peqGold RNAPure extraction solution (Peqlab

Biotechnology, Erlangen) and total RNA was extracted following the manufacturer's instructions. Finally, the RNA was dissolved in DEPC (diethylpyrocarbonate)-H_2_O and stored at -80°C until further use. After incubation with DNase (Promega-Kit RQ1) a DNA-free RNA sample was obtained. To confirm the absence of remaining DNA in the DNase digested RNA samples, a PCR-reaction was performed using HotStarTaq (Qiagen, Hilden) and selected gene specific oligonucleotide primers (see additional file 10 probes for southern blotting primers *lrp*). Only RNA, which did not yield any product after amplification (40 cycles) was used in subsequent RT-PCR's. RNA integrity was proven by using the 2100 Bioanalyzer (Agilent Technologies, Waldbronn) or alternatively with denaturating 1% TBE-agarose-gels containing 20 mM guanidinium thiocyanate.

### Microarray analysis

Wild type R1 and the deletion strains (Δ*lrp*, Δ*lrpA1*) utilized for the microarray approach were grown in complex medium. Total RNA (5 μg), isolated from cells having an OD_600 _of 0.4 (4 × 10^8 ^cells/ml), was reverse transcribed into Cy3/Cy5-labeled cDNA using CyScribe First-Strand cDNA Synthesis Kit with enclosed random nonamer primers and Cy3-/Cy5-dUTP (both Amersham Biosciences, Freiburg). Labelled cDNA was hybridized to in-house fabricated whole genome DNA-microarrays [55] at 64°C overnight. To determine the fluorescence ratios the slides were scanned (GenePix 4000 B, Axon Instruments) and the data were extracted using the GenePix Pro 6 software. After background substraction, pin-wise normalization and data evaluation by a Student's T-test, those transcripts displaying a p-value equal or lower than 5.10^-5 ^and a ratio of +/- 1.3 were selected as significantly regulated. A detailed description of the microarray design, experimental procedure and data-evaluation is described in [55]. We considered ratios with a p-value equal or lower than 5 × 10^-5 ^as significant. This reflects a stringent interpretation of data as a two times less stringent p-value results in 4.8% false positives [56]. The data obtained from the microarray experiment were deposited at http://www.ebi.ac.uk/miamexpress under the accession number (E-MEXP-1447).

### Reverse transcription-quantitative PCR and RACE

5 μg DNA-free total RNA was reverse transcribed using 0.5 μg random hexamer primers (Promega, Mannheim) and Superscript III reverse transcriptase (Invitrogen, Karlsruhe) according to the manufacturer's instructions. 1 μ1 of the cDNA reaction mixture was quantified by using the SYBR Green PCR Master Mix Kit (Applied Biosystems, Darmstadt) in a GeneAmp 5700 Sequence Detection System (Applied Biosystems, Darmstadt) in a final reaction volume of 25 μl. The primer pairs (additional file 10) for amplification were designed with Primer Express 2.0 (Applied Biosystems, Darmstadt) and were added to a final concentration of 0.2 μM. The data were analyzed via the 2^ΔΔCt^-method using the mean-C_t_-value of 3 replicate reactions per primer pair. The constitutively expressed gene OE4759F, encoding a S-layer glycoprotein, was chosen as internal standard. RACE (rapid amplification of cDNA ends) was essentially performed by an RNA circularization mediated method according to [34] to determine the 5'ends and the 3'-ends of transcripts.

### Northern blot hybridizations

15 μg total RNA was separated by electrophoresis on 1% TBE-agarose-gel, containing 20 mM guanidinium thiocyanate. Gels were then equilibrated in alkaline buffer [57] and transferred onto Hybond N^+ ^membrane (GE-Healthcare, München), by vacuum blotting for 3 hours. For generation of DIG-dUTP-labelled RNA-probes a PCR amplified DNA-fragment (additional file 10), including the T7-promoter sequence was generated in order to function as a template for *in vitro *transcription with T7-RNA-polymerase (DIG RNA labelling kit (SP6/T7), Roche Applied Science, Mannheim). Furthermore, hybridization and chemiluminiscent detection were carried out using the "DIG Wash and Block Buffer Set" (Roche Applied Science, Mannheim) according to "DIG system user's guide for filter hybridization" (Boehringer, Mannheim). All oligonucleotides used for generating the probes are mentioned in additional file 10.

### Expression and purification of LrpA1

*LrpA1 *was amplified by PCR using oligonucleotides (additional file 10) including the sequence for the restriction sites of *NdeI *and *XhoI*. After digestion with these enzymes the PCR fragment was cloned into the vector pET26b (Novagen, Darmstadt). The obtained LrpA1-expression vector was transformed into *E. coli *BL21(DE3). The expressed polypeptide contained a C-terminal His_6_-tag. A single colony was picked in order to inoculate 30 ml Luria-Bertani (LB) medium supplemented with 50 μg/ml kanamycin. The culture was grown overnight in a rotary shaker at 37°C. On the next day the culture was used to inoculate 1 liter of the identical medium also supplemented with 50 μg/ml kanamycin. Protein expression was induced with 0.6 mM isopropyl-β-D-thiogalactopyranoside (IPTG) at an OD_600 _of 0.8. The cells were harvested after 3 hours of growth by centrifugation for 10 min at 5000 g (4°C). The pellet was resuspended in 30 ml buffer A (8 M urea; 100 mM NH_2_PO_4_; 10 mM Tris-HCl, pH 8.0) with 10 mM imidazole and disrupted by sonification (3 × 15 sec; 50% duty cycle; Branson Sonifier). The lysate was centrifuged for 80 min at 50000 g. Subsequently, 1.5 ml of Ni-NTA fast flow matrix (Qiagen, Hilden) was added to the supernatant and was incubated on a rotary wheel for 2 h (4°C). Afterwards the Ni-NTA matrix was packed on a column, washed three times with 7.5 ml of buffer A plus 20 mM imidazole and eluted in three times 1.5 ml fractions with buffer A plus 150 mM imidazole. To restore the native conditions the purified LrpA1-His_6 _was dialyzed against cell-free extract (CFE) buffer (3 M KCl; 1 M NaCl; 10 mM HEPES, pH 7.1) over night at room temperature. To prove successful refolding we performed CD spectroscopy (method see below). For the determination of multimerisation, we performed size exclusion chromatography by using a 3.2/3 Superdex 200 column (GE-Healthcare, München) on a SMART chromatography device (GE-Healthcare, München) (flow-rate 50 μl per min CFE buffer). Presence of protein was detected at 280 nm.

### Circular dichroism spectroscopy

To determine the secondary structures of LrpA1 after renaturation (see Expression and purification of LrpA1 (Methods)) we used circular dichroism in the far-UV (190-250 nm). CD-Spectra were monitored using a JASCO-J-810 spectrometer. After renaturation against the high salt CFE buffer (3 M KCl; 1 M NaCl; 5 mM MgCl_2_; 10 mM HEPES, pH 7.1) LrpA1 had a final protein concentration of 1.2 mg/ml and was measured in 0.01 mm quartz cuvettes (Helma). All measurements were performed in CFE buffer at 21°C. The spectra were calculated from the average of 12 scans and repeated in two independent measurements, followed by the subtraction of spectra measured only with CFE buffer. Percentages of secondary structure were determined with the CDNN-program [58]. For secondary structure prediction we used the program "Scratch Protein Predictor"(Expasy).

### Protein-DNA-binding assay for halophilic proteins

Analytical DNA-affinity chromatography was performed by a modification of the method described by [59]. For each experiment, 150 μl of streptavidin sepharose high performance (GE-Healthcare, München) suspension was spun down at 340 g in a column (MoBiTec M1002S, Göttingen) to remove ethanol. After five consecutive washing steps (500 μl of binding buffer: 0.15 M NaCl; 20 mM Na_2_PO_4_, pH 7.5) streptavidin sepharose was incubated with biotin-labelled DNA probes (61 pmol) for 2 h at room temperature with gentle shaking. Biotinylated DNA was prepared by PCR using biotin-labelled primers (Metabion, Martinsried) for amplification of the *lrpA1 *and the aspartate transaminase (*aspB3*) promoter, as well as a fragment of the *flgB1 *gene as negative-control. For binding of LrpA1 to the *aspB3 *promoter, reactions were performed in CFE buffer (3 M KCl; 1 M NaCl; 5 mM MgCl_2_; 10 mM HEPES, pH 7.1) in the presence of either 5 mM L-aspartate or 5 mM L-arginine. Columns were washed three times with CFE buffer. Unbound DNA was monitored spectrophotometrically. Protein-DNA-binding reactions were carried out by incubation of LrpA1 with the DNA-affinity matrix at room temperature for 4 h with shaking. We applied a stoichiometric excess of protein (2 nmol) relative to the molar amount of DNA (61 pmol). The mixture was then transferred to a column, centrifuged and washed twice with 200 μl CFE. LrpA1 was eluted from the DNA-Sepharose with 100 μl of 1% SDS and analyzed by SDS-PAGE (NuPAGE^®^Pre-Cast Gel System, Invitrogen) with subsequent silver staining. We performed three independent binding experiments. From these data we quantified band intensities in each single gel using densitometry (Total Lab Version 1.11). Both, monomeric as well as dimeric bands account for the depicted final values. The band with the highest intensity in each gel represents 100% (see Fig 4A lane 2, Fig 4B lane 3, Fig 4C lane 3). The two other bands with weaker binding intensity were calculated in relation to 100% highest intensity. Oligonucleotides used for the amplification of the DNA fragments are mentioned in additional file 10.

## Authors' contributions

RS performed most of the experiments described in this article (microarray experiments, determination of the transcriptional start sites and 3'UTR, Northern hybridzations, DNA-binding assays) and wrote the manuscript with support of DO as the supervisor of this study. CS constructed the deletion mutants (Δ*lrpA1*, Δ*lrp*) and KF the overexpression strains (↑*lrpA1*, ↑lrp). VT developed the halophilic DNA-binding assay and AW participated in establishing the Microarray technique for *H. salinarum *R1 in our department. All authors carefully read the manuscript.

## Supplementary Material

Additional file 1 **Structure-based sequence alignment of the *H. salinarum *LrpA1 (OE2621R) with other archaeal and bacterial Lrp-homologues**. The percentages in parenthesis represent a sequence comparison between LrpA1 and the aligned sequences. The alignment includes *P. furiosus *LrpA (PF1601; 38%), *M. jannaschii *Ptr2 (MJ0723; 30%), *H. walsbyi *Lrp-like protein (HQ3354A; 76%), *H. salinarum *Lrp (OE3923F; 25%), *S. solfataricus *LysM (SSO0157; 21%), *B. subtilis *LrpC (BSU04250; 24%), *E. coli *AsnC (APECO1_2720; 26%), and *E. coli *Lrp (b0889; 23%). The HTH DNA-binding motif (αB-αC) and the RAM-domain (β2αDβ3β4αEβ5) are boxed, including the asparagine binding site of *E. coli *AsnC. Amino acids are shaded in grey according to sequence conservation. Conserved methionine/prolines of the LrpA1-subgroup are shaded in blue. LrpA1 shares highest sequence identity (76%) with the Lrp-like regulator (HQ3354A) from *Haloquadratum walsbyi*. A comparison between LrpA1 and other Lrp-homologues revealed 38% identity with LrpA (PF1601) from *P. furiosus*, 30% identity with *Ptr2 *(MJ0723) from *M. jannaschii*, 21% identity with *S. solfataricus *LysM (SSO0157) and 24% identity with LrpC (BSU04250) from *Bacillus subtilis. E. coli *Lrp (b0889) showed 23% and *E. coli *AsnC (APECO1_2720) 26% identity. Secondary structure elements are indicated as red α-helices and green β-strands. In both *H. salinarum *Lrp proteins the N-terminal helix-turn-helix (HTH) motif and the C-terminal regulation of amino acid metabolism (RAM)-domain were identified based on the structure of cristallized Lrp/AsnC homologues. The figure was made by using the INDONESIA alignment package (D. Madsen, P. Johansson and G.J. Kleywegt manuscript in preparation). Click here for file

Additional file 2 **Growth curves of Δ*lrp*, Δ*lrpA1*, ↑*lrp *and ↑*lrpA1***. Growth curves of the deletion strains Δ*lrp *and Δ*lrpA1 *as well as the overexpressing strains ↑*lrp *and ↑*lrpA1 *were compared to the wild type strain R1. All strains were grown in complex medium. Growth occurred aerobic in the dark for the deletion strains (A) and anaerobic in the light for the overexpression strains (B). The optical density of the cultures was determined at OD_600_. Click here for file

Additional file 3 **Southern blot analysis to verify the correct genotype of the deletion mutants (Δ*lrpA1 *Δ*lrp*)**. In a first approach deletion strains were pre-selected by PCR (oligonucleotides see additional file 10; probes for southern blotting). We used two primer pairs, one, which amplifies the entire gene to be deleted (only in wild type, not in the mutant) and another, which anneals to flanking regions up-and downstream of that ORF. Typically a mutant strain does not yield the first, but the second product of the size: (wild type amlicon-gene length). As a positive control we have used chromosomal DNA of wild type cells. To ensure that the deleted gene has not been relocated by chromosomal rearrangements we subsequently confirmed the mutant genotype by southern blotting. Genomic DNA from the PCR-positive clones as well as wild type DNA was cut with the restriction enzyme *BglI*. Southern blot hybridization was performed using two different types of digoxygenin labelled probes generated by PCR. (see additional file 10: flanking probe (Δ*lrpA1*, Δ*lrp*) and gene probe (*lrpA1*, *lrp*)). Samples were separated by denaturing agarose gel electrophoresis (1%), and vacuum blotted onto a nylon membrane. Hybridization and detection were performed with "DIG Easy Hyb" (Roche Diagnostics) according to the manufacturer's instructions. The obtained fragments are marked by an arrow (A1, 2 and B1, 2) and explained in additional table S1. Figure A shows the southern blot for the PCR-positive deletion strains Δ*lrpA1*. PCR-positive clones (lane 1-9), wild type DNA (lane 11) and a Dig-labelled DNA-standard (lane 12). Using two different types of probes (flanking probe A1 and gene probe A2) fragments obtained from southern blot are marked by an arrow and described in additional table S1. PCR-positive deletion strains Δ*lrp *(lane 1-3), wild type DNA (4, 5) and a Dig-labelled DNA-standard (lane 7) were loaded to a 1% agarose gel (B). The resulted fragments after using the flanking probe (B1) and the gene probe (B2) are marked by an arrow and corresponding sizes are shown in additional table S1. Click here for file

Additional file 4 **Differentially expressed genes in Δ*lrpA1***. All significantly differentially expressed genes in Δ*lrpA1 *having a ratio higher than +/-1.7 and those between +/-1.7 and +/-1.3 are depicted in this table. The regulated genes are sorted by their Identification number (ID). Click here for file

Additional file 5 **RT-qPCR data compared to microarray data in Δ*lrpA1 *compared against wild type R1**. Additional table S2 shows a comparison of RT-qPCR data with the microarray data. Total RNA was isolated from the deletion mutant Δ*lrpA1 *and wild type at a cell density OD_600 _0.4. We determined the transcript amount of the genes *aspB3 *and OE6130F, which encodes for a conserved hypothetical protein. Click here for file

Additional file 6 **Expression, oligomerization and folding of LrpA1**. Heterologous expression of the His_6_-tagged *H. salinarum *LrpA1 in *E. coli *BL21(DE3) and protein purification analyzed by SDS-PAGE. *E. coli *extracts before induction (lane 1), two and three hours after induction with 0.6 mM IPTG (lane 2, 3) and purified protein, displayed by an arrow (lane 4-6) (A). After dialysis against a high salt buffer correct folding of LrpA1 was proved by CD-spectroscopy, were 56% α-helices, 11% β-sheet, 14% β-turn and 24% random coil structure was determined (B). The theoretical calculated values for LrpA1 are 42% α-helices, 27% β-sheet and 31% random coil structure was determined. Folded LrpA1 in high salt has a predominant α-helical structure. An aberrance of ~10% between the measured and the theoretical value is in the range of error and has been shown in previous studies [60] (B). The size exclusion chromatography elution profile showed dimerisation of LrpA1 after renaturation (C). Calibration standards used for this run are indicated in additional table S2 (C). LrpA1 elutes at a volume of 1.32 ml which is a corresponding molecular weight 31.1 kDa showing a LrpA1 dimer. The theoretical size of a LrpA1 monomer is 15.2 kDa. Click here for file

Additional file 7 **Sequence comparison of the three *H. salinarum *aspartate transaminases**. AspB1, AspB2 and AspB3 were compared with aspartate transaminases subgroup Ia (having a conserved R at the position marked by an □) and aspartate transaminases Ib (having an conserved K at the position marked by an x). Click here for file

Additional file 8 **Western blot analysis to detect the overexpression of *lrp *on protein level**. Transcription of the *lrp *gene was under the control of the bacteriorhodopsin (bop) promoter resulting in the KF203 (↑*lrp*) mutant. The *bop *promoter is maximally induced under light exposure and anaerobic conditions. Therefore wild type cells and cells from the Lrp-overexpression strain were grown anaerobically under light exposure and harvested at an OD_600 _of 0.8. For detection of the Lrp protein we used an antibody against Lrp. Proteins from strains as indicated in the figure were separated on a gradient gel (4-12%), blotted on a nitrocellulose membrane and finally subjected to an immune detection reaction with an antibody against Lrp. Low expression was observed for the wild type (lane 1), whereas significant overexpression was detected in the Lrp-overexpression strain (lane 2). Furthermore we tested the Δ*lrp *deletion mutant, grown aerobically in the dark. Using an antibody against Lrp (lane 3) no signal could be obtained, as expected. Click here for file

Additional file 9 **Differentially expressed genes in Δ*lrp***. All significantly differentially expressed genes in Δ*lrp *having a ratio higher than +/-1.7 and those between +/-1.7 and +/-1.3 are depicted in this table. The regulated genes are sorted by their Identification number (ID). Click here for file

Additional file 10 **A table of oligonucleotides**. Oligonucleotides used in these experiments Click here for file

Additional file 11 **Scheme of integration of pKF204 into the genome of *H. salinarum *resulting in KF204 (↑*lrpA1*) mutant**. The plasmid pKF204 contains a portion of the 5'end of the *lrpA1 *gene. The *bop *promoter P*bop *(violet box) was inserted upstream of the truncated *lrpA1 *gene (black arrow). The plasmid contains a selection marker (Mev^R^). After integration of pKF204 into the *H. salinarum *genome, only the *lrpA1 *gene downstream the *bop *promoter is functionally transcribed, while transcription product under the native promoter is truncated and presumably not functional. The KF203 (↑lrp) mutant was constructed similarly using plasmid pKF203. Click here for file
