# Supplementary figures and images for: Transcriptional control by two leucine-responsive regulatory proteins in Halobacterium salinarum R1
Source: BMC Mol Biol. 2010 May 28;11:40. doi: 10.1186/1471-2199-11-40 (PMC2894021; doi:10.1186/1471-2199-11-40)

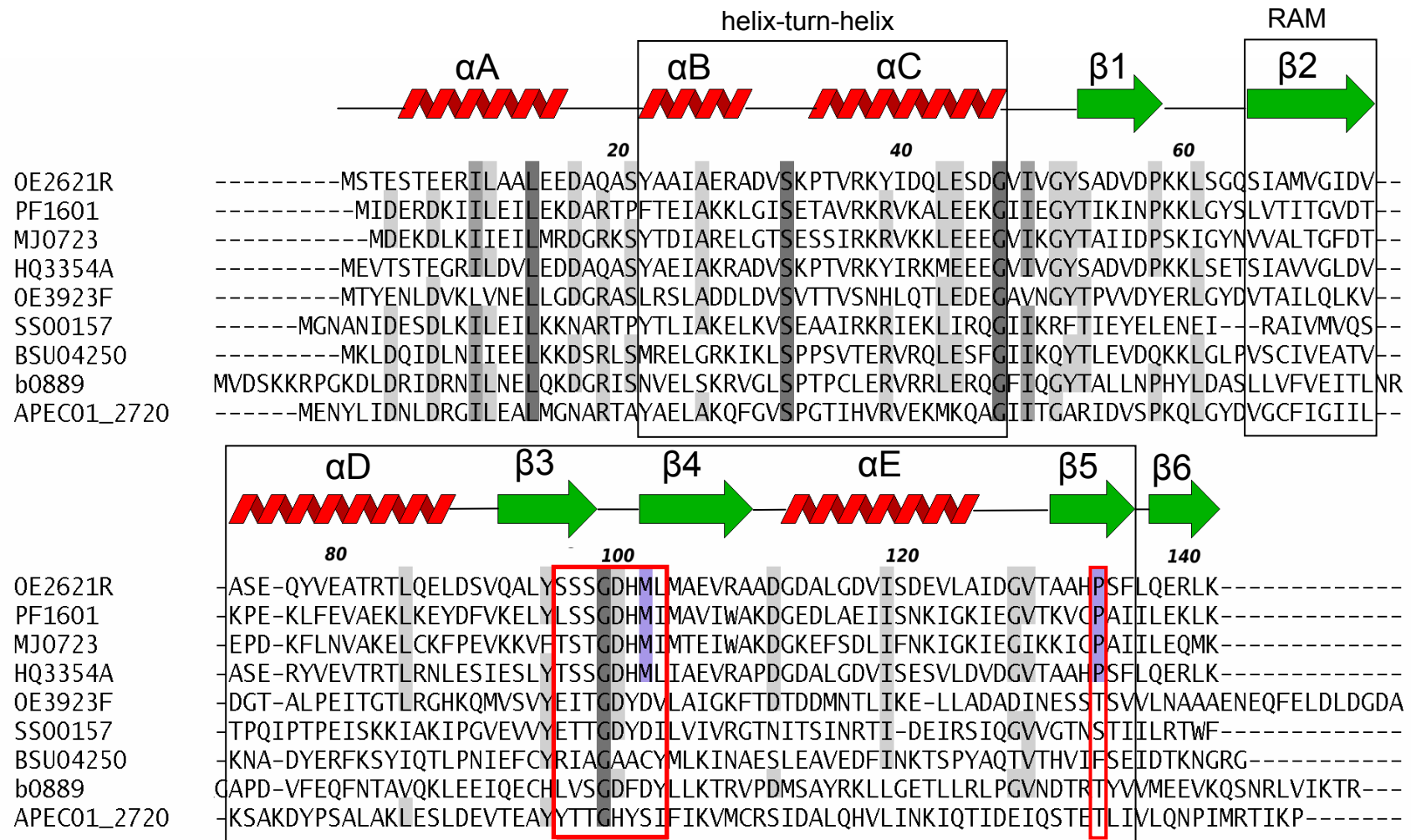

Supplement: Additional file 1 — Structure-based sequence alignment of the H. salinarum LrpA1 (OE2621R) with other archaeal and bacterial Lrp-homologues. The percentages in parenthesis represent a sequence comparison between LrpA1 and the aligned sequences. The alignment includes P. furiosus LrpA (PF1601; 38%), M. jannaschii Ptr2 (MJ0723; 30%), H. walsbyi Lrp-like protein (HQ3354A; 76%), H. salinarum Lrp (OE3923F; 25%), S. solfataricus LysM (SSO0157; 21%), B. subtilis LrpC (BSU04250; 24%), E. coli AsnC (APECO1_2720; 26%), and E. coli Lrp (b0889; 23%). The HTH DNA-binding motif (αB-αC) and the RAM-domain (β2αDβ3β4αEβ5) are boxed, including the asparagine binding site of E. coli AsnC. Amino acids are shaded in grey according to sequence conservation. Conserved methionine/prolines of the LrpA1-subgroup are shaded in blue. LrpA1 shares highest sequence identity (76%) with the Lrp-like regulator (HQ3354A) from Haloquadratum walsbyi. A comparison between LrpA1 and other Lrp-homologues revealed 38% identity with LrpA (PF1601) from P. furiosus, 30% identity with Ptr2 (MJ0723) from M. jannaschii, 21% identity with S. solfataricus LysM (SSO0157) and 24% identity with LrpC (BSU04250) from Bacillus subtilis. E. coli Lrp (b0889) showed 23% and E. coli AsnC (APECO1_2720) 26% identity. Secondary structure elements are indicated as red α-helices and green β-strands. In both H. salinarum Lrp proteins the N-terminal helix-turn-helix (HTH) motif and the C-terminal regulation of amino acid metabolism (RAM)-domain were identified based on the structure of cristallized Lrp/AsnC homologues. The figure was made by using the INDONESIA alignment package (D. Madsen, P. Johansson and G.J. Kleywegt manuscript in preparation). [file 1471-2199-11-40-S1.PDF]

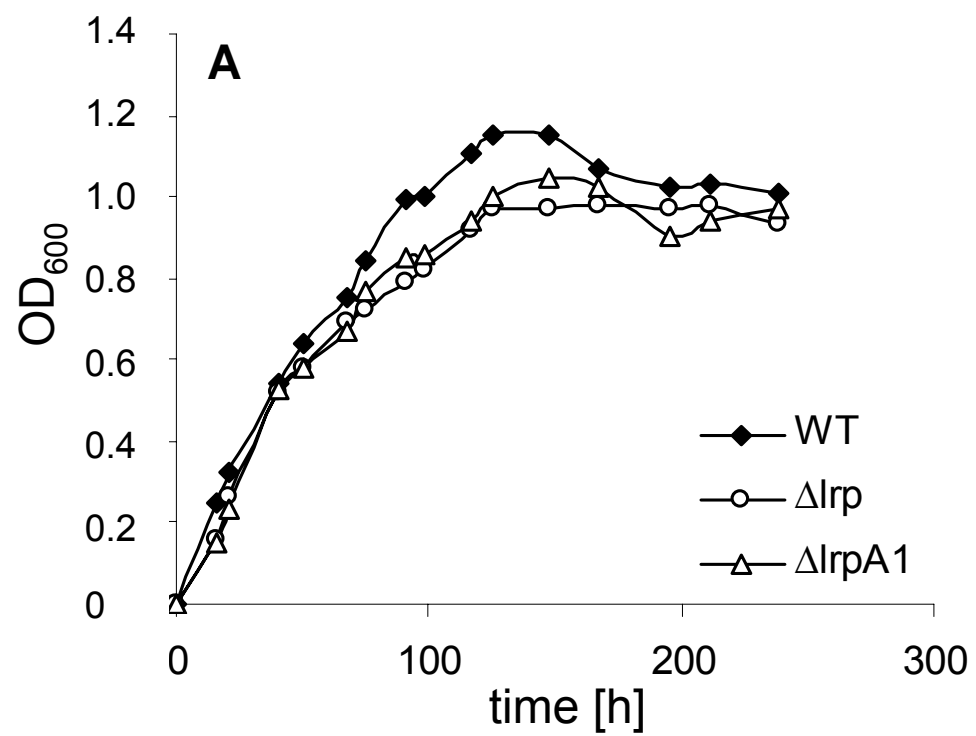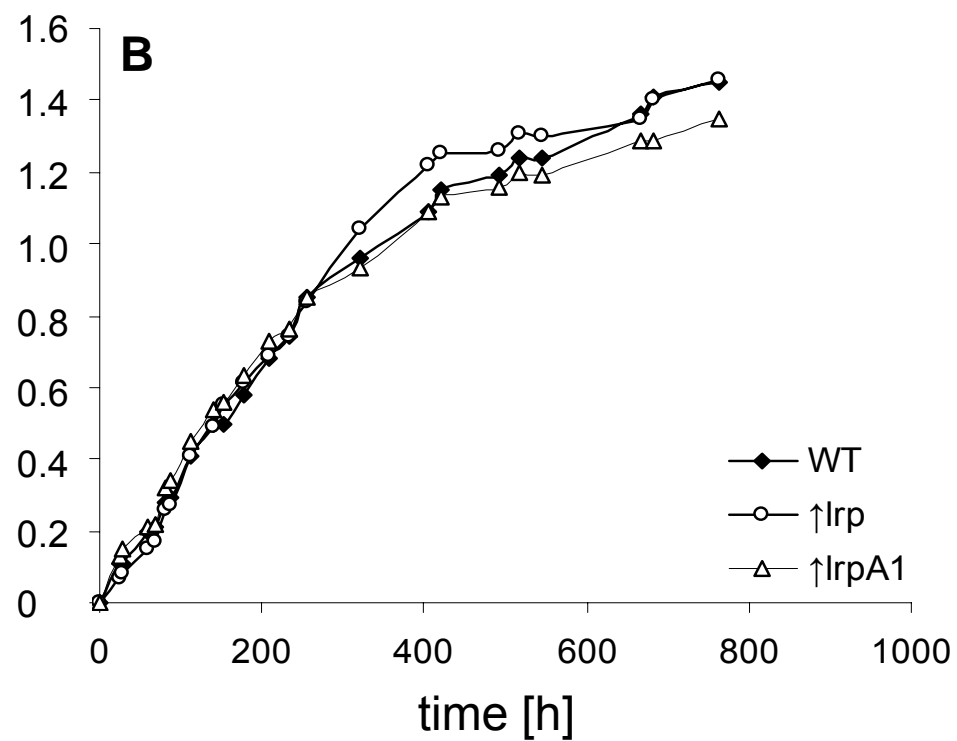

Supplement: Additional file 2 — Growth curves of Δlrp, ΔlrpA1, ↑lrp and ↑lrpA1. Growth curves of the deletion strains Δlrp and ΔlrpA1 as well as the overexpressing strains ↑lrp and ↑lrpA1 were compared to the wild type strain R1. All strains were grown in complex medium. Growth occurred aerobic in the dark for the deletion strains (A) and anaerobic in the light for the overexpression strains (B). The optical density of the cultures was determined at OD600. [file 1471-2199-11-40-S2.PDF]

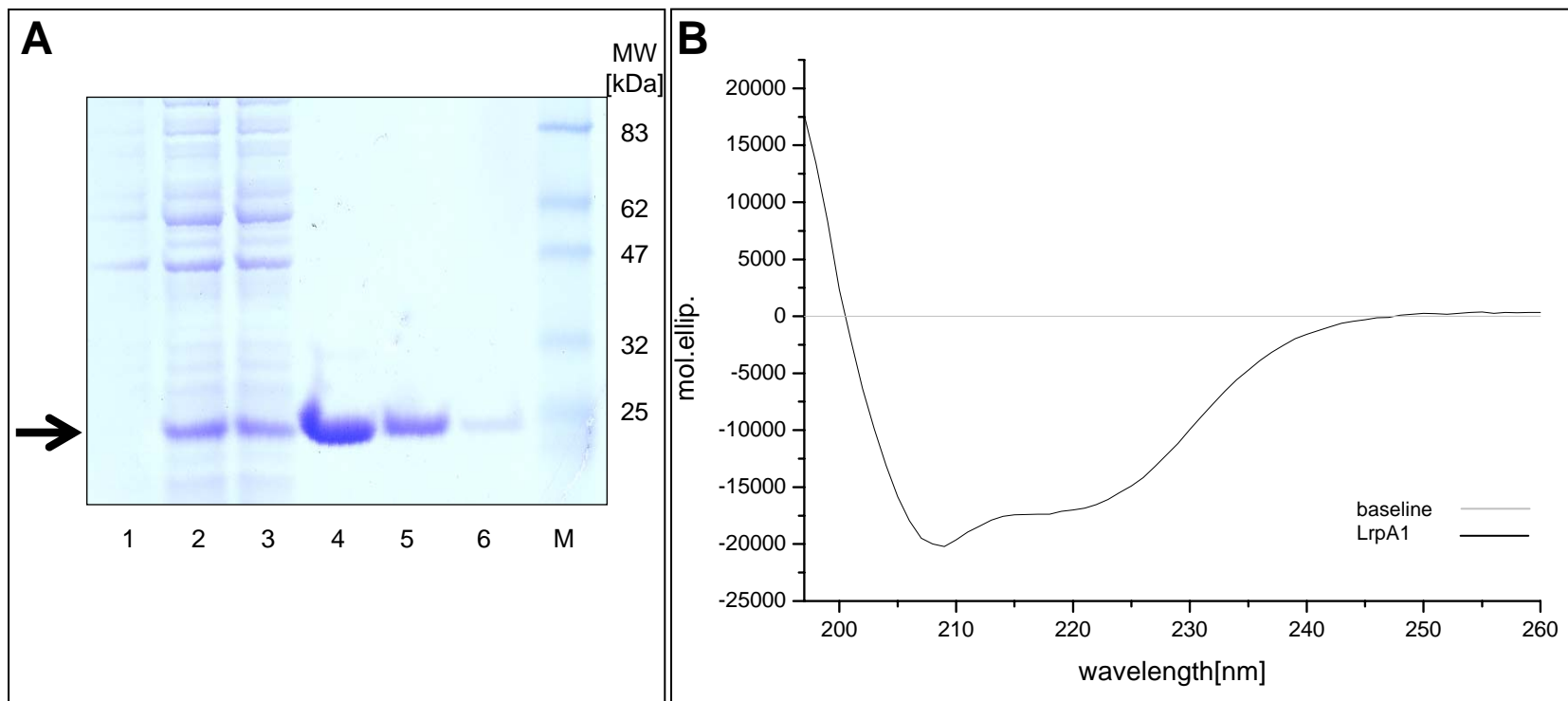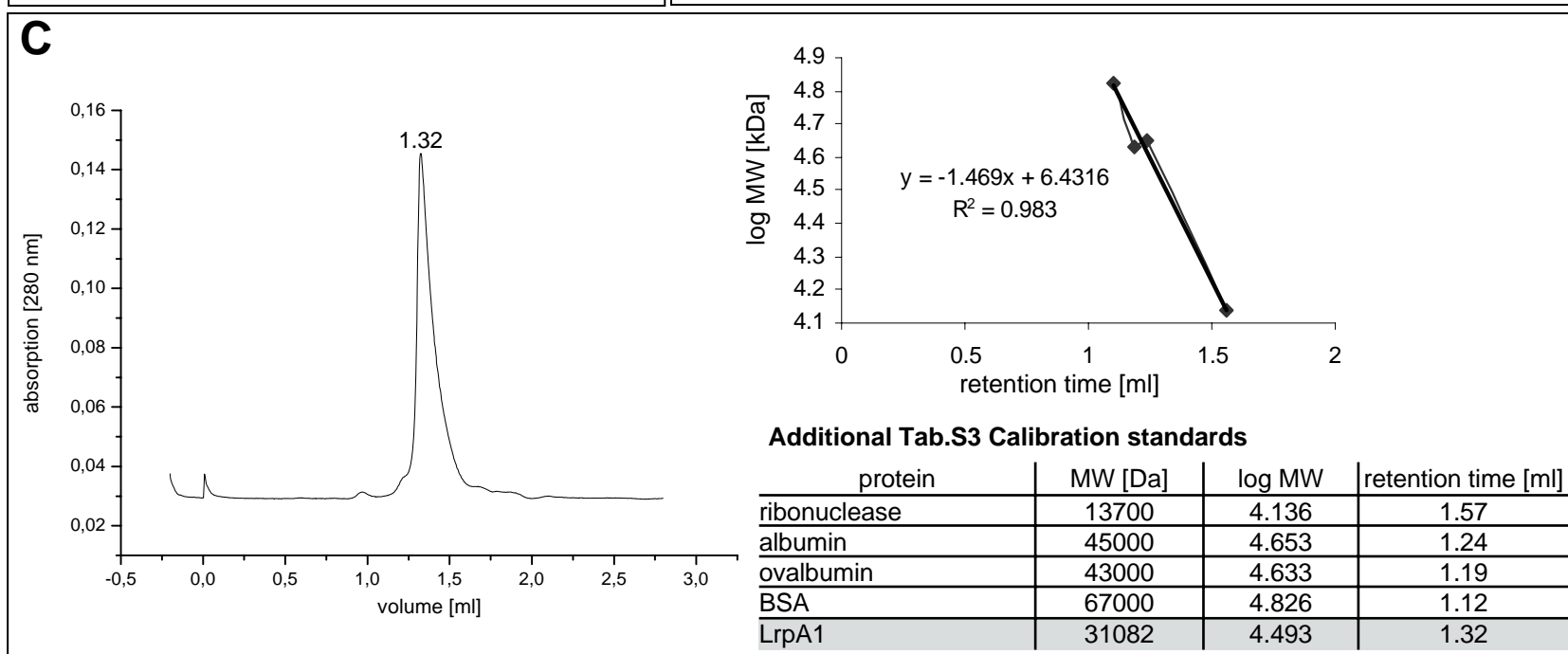

Supplement: Additional file 6 — Expression, oligomerization and folding of LrpA1. Heterologous expression of the His6-tagged H. salinarum LrpA1 in E. coli BL21(DE3) and protein purification analyzed by SDS-PAGE. E. coli extracts before induction (lane 1), two and three hours after induction with 0.6 mM IPTG (lane 2, 3) and purified protein, displayed by an arrow (lane 4-6) (A). After dialysis against a high salt buffer correct folding of LrpA1 was proved by CD-spectroscopy, were 56% α-helices, 11% β-sheet, 14% β-turn and 24% random coil structure was determined (B). The theoretical calculated values for LrpA1 are 42% α-helices, 27% β-sheet and 31% random coil structure was determined. Folded LrpA1 in high salt has a predominant α-helical structure. An aberrance of ~10% between the measured and the theoretical value is in the range of error and has been shown in previous studies [60] (B). The size exclusion chromatography elution profile showed dimerisation of LrpA1 after renaturation (C). Calibration standards used for this run are indicated in additional table S2 (C). LrpA1 elutes at a volume of 1.32 ml which is a corresponding molecular weight 31.1 kDa showing a LrpA1 dimer. The theoretical size of a LrpA1 monomer is 15.2 kDa. [file 1471-2199-11-40-S6.PDF]

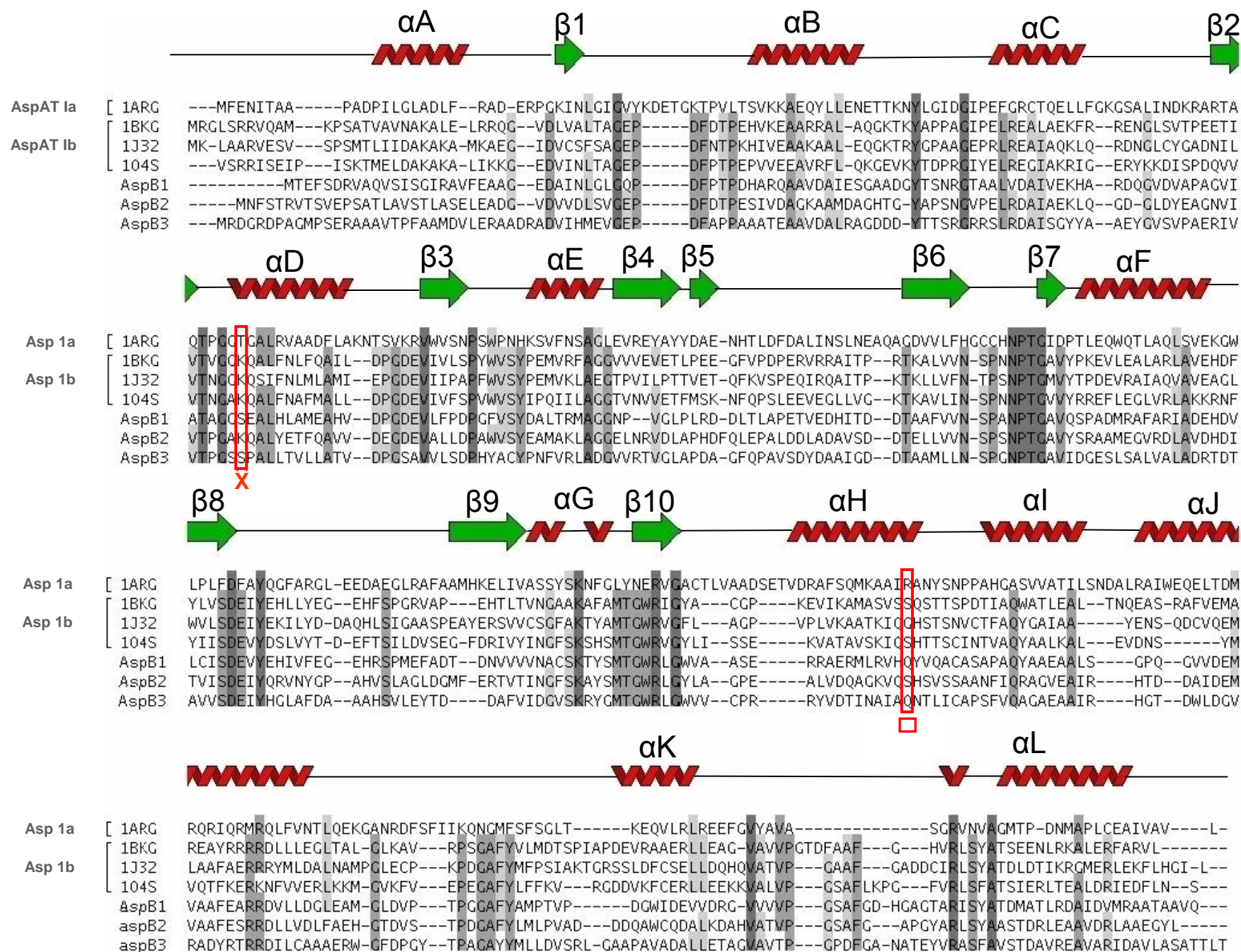

Supplement: Additional file 7 — Sequence comparison of the three H. salinarum aspartate transaminases. AspB1, AspB2 and AspB3 were compared with aspartate transaminases subgroup Ia (having a conserved R at the position marked by an □) and aspartate transaminases Ib (having an conserved K at the position marked by an x). [file 1471-2199-11-40-S7.PDF]

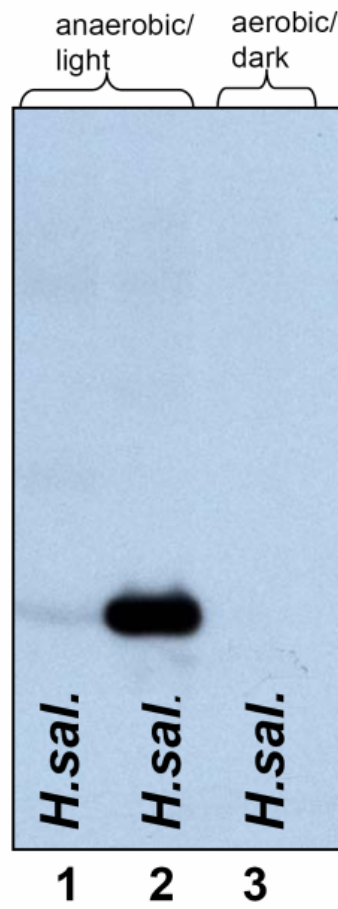

Supplement: Additional file 8 — Western blot analysis to detect the overexpression of lrp on protein level. Transcription of the lrp gene was under the control of the bacteriorhodopsin (bop) promoter resulting in the KF203 (↑lrp) mutant. The bop promoter is maximally induced under light exposure and anaerobic conditions. Therefore wild type cells and cells from the Lrp-overexpression strain were grown anaerobically under light exposure and harvested at an OD600 of 0.8. For detection of the Lrp protein we used an antibody against Lrp. Proteins from strains as indicated in the figure were separated on a gradient gel (4-12%), blotted on a nitrocellulose membrane and finally subjected to an immune detection reaction with an antibody against Lrp. Low expression was observed for the wild type (lane 1), whereas significant overexpression was detected in the Lrp-overexpression strain (lane 2). Furthermore we tested the Δlrp deletion mutant, grown aerobically in the dark. Using an antibody against Lrp (lane 3) no signal could be obtained, as expected. [file 1471-2199-11-40-S8.PDF]

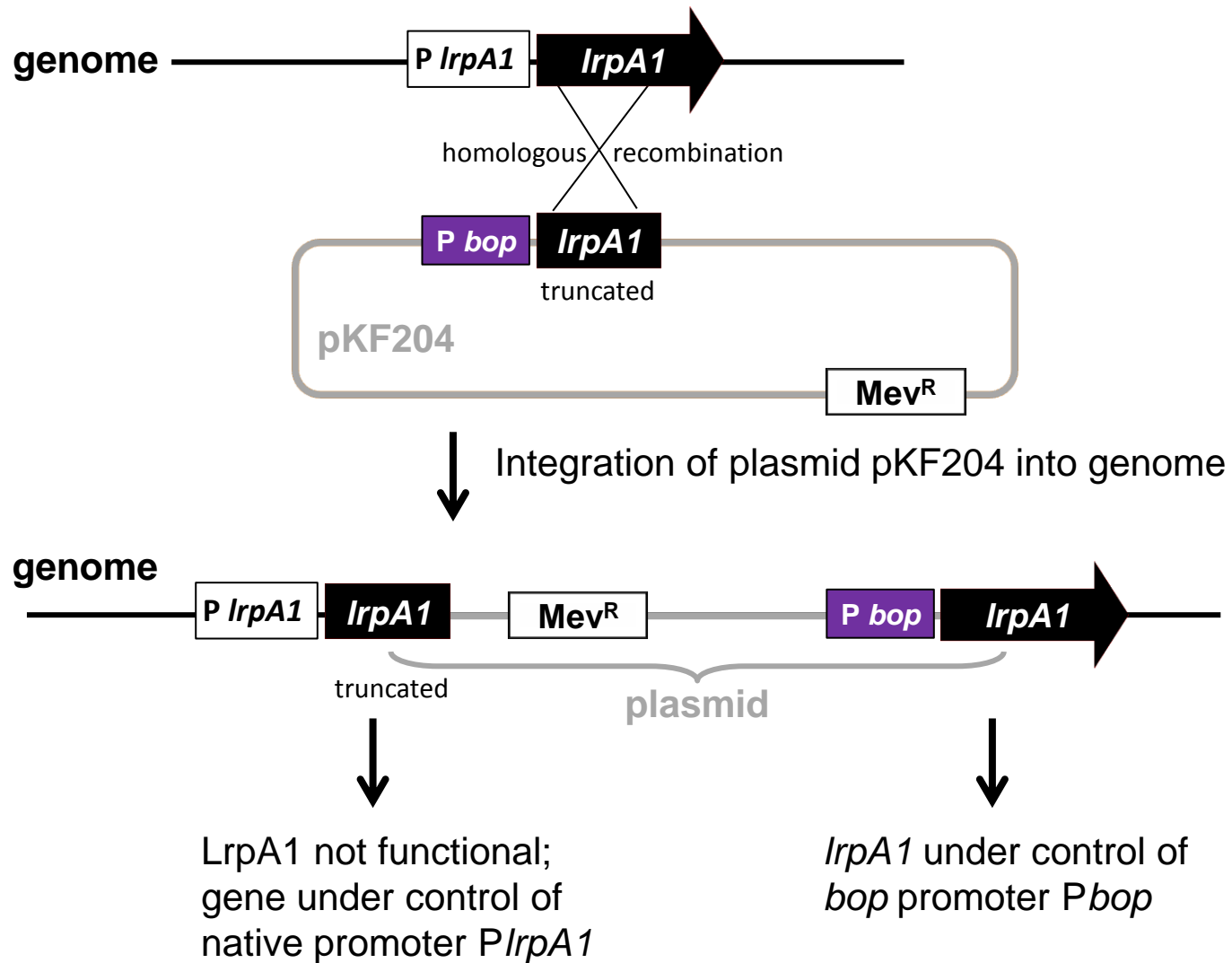

Supplement: Additional file 11 — Scheme of integration of pKF204 into the genome of H. salinarum resulting in KF204 (↑lrpA1) mutant. The plasmid pKF204 contains a portion of the 5'end of the lrpA1 gene. The bop promoter Pbop (violet box) was inserted upstream of the truncated lrpA1 gene (black arrow). The plasmid contains a selection marker (MevR). After integration of pKF204 into the H. salinarum genome, only the lrpA1 gene downstream the bop promoter is functionally transcribed, while transcription product under the native promoter is truncated and presumably not functional. The KF203 (↑lrp) mutant was constructed similarly using plasmid pKF203. [file 1471-2199-11-40-S11.PDF]
