# Supplementary material for: Transcriptional control by two leucine-responsive regulatory proteins in Halobacterium salinarum R1
Source: BMC Mol Biol. 2010 May 28;11:40. doi: 10.1186/1471-2199-11-40 (PMC2894021; doi:10.1186/1471-2199-11-40)
Supplement: Additional file 3 — Southern blot analysis to verify the correct genotype of the deletion mutants (ΔlrpA1 Δlrp). In a first approach deletion strains were pre-selected by PCR (oligonucleotides see additional file 10; probes for southern blotting). We used two primer pairs, one, which amplifies the entire gene to be deleted (only in wild type, not in the mutant) and another, which anneals to flanking regions up-and downstream of that ORF. Typically a mutant strain does not yield the first, but the second product of the size: (wild type amlicon-gene length). As a positive control we have used chromosomal DNA of wild type cells. To ensure that the deleted gene has not been relocated by chromosomal rearrangements we subsequently confirmed the mutant genotype by southern blotting. Genomic DNA from the PCR-positive clones as well as wild type DNA was cut with the restriction enzyme BglI. Southern blot hybridization was performed using two different types of digoxygenin labelled probes generated by PCR. (see additional file 10: flanking probe (ΔlrpA1, Δlrp) and gene probe (lrpA1, lrp)). Samples were separated by denaturing agarose gel electrophoresis (1%), and vacuum blotted onto a nylon membrane. Hybridization and detection were performed with "DIG Easy Hyb" (Roche Diagnostics) according to the manufacturer's instructions. The obtained fragments are marked by an arrow (A1, 2 and B1, 2) and explained in additional table S1. Figure A shows the southern blot for the PCR-positive deletion strains ΔlrpA1. PCR-positive clones (lane 1-9), wild type DNA (lane 11) and a Dig-labelled DNA-standard (lane 12). Using two different types of probes (flanking probe A1 and gene probe A2) fragments obtained from southern blot are marked by an arrow and described in additional table S1. PCR-positive deletion strains Δlrp (lane 1-3), wild type DNA (4, 5) and a Dig-labelled DNA-standard (lane 7) were loaded to a 1% agarose gel (B). The resulted fragments after using the flanking probe (B1) and the gene probe (B2) [file 1471-2199-11-40-S3.PDF]

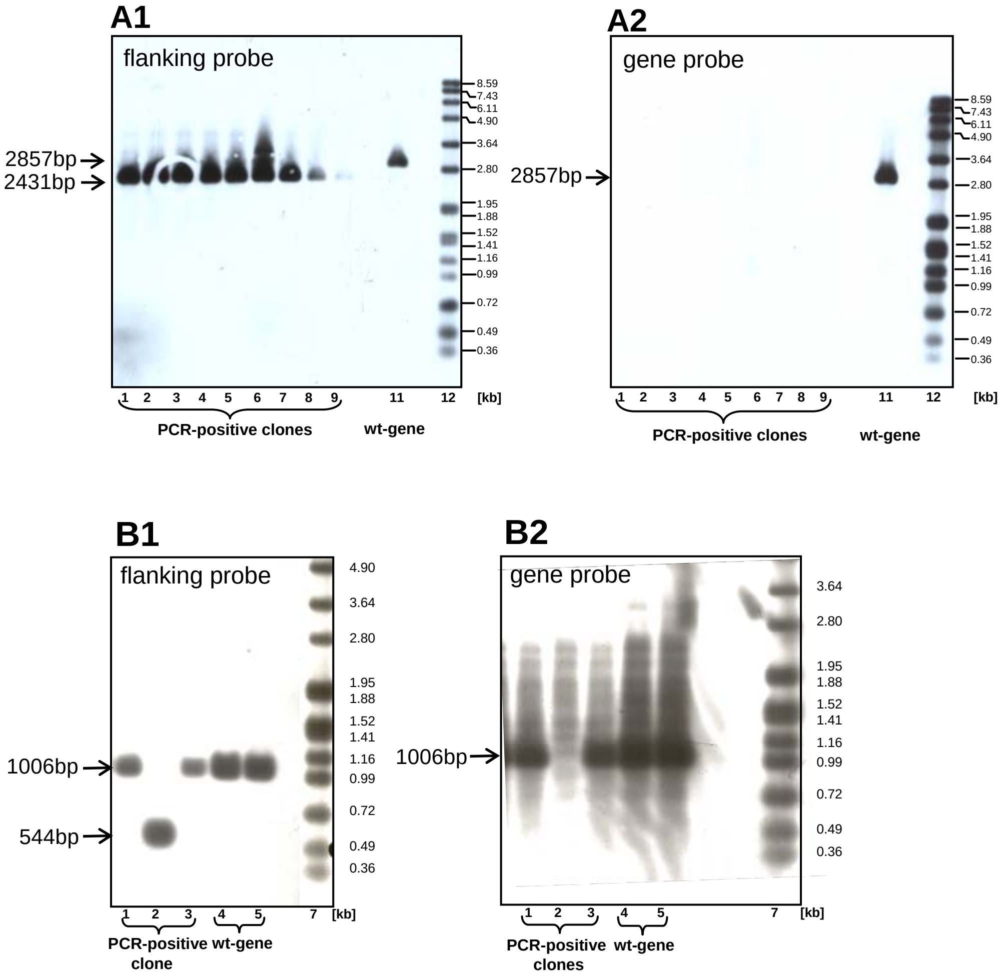

Additional Tab. S1 Description of the fragment sizes

gene	size [bp]	wt-fragment [bp]	fig.	deletion-mutant	deletion-fragment [bp]	fig.
lrpA1 (OE2621R)	426	2857	A2	*ΔlrpA1*	2431	A1
lrp (OE3932F)	46	1006	B2	*Δlrp*	544	B1
