# Supplementary material for: Transcriptional control by two leucine-responsive regulatory proteins in Halobacterium salinarum R1
Source: BMC Mol Biol. 2010 May 28;11:40. doi: 10.1186/1471-2199-11-40 (PMC2894021; doi:10.1186/1471-2199-11-40)
Supplement: Additional file 4 — Differentially expressed genes in ΔlrpA1. All significantly differentially expressed genes in ΔlrpA1 having a ratio higher than +/-1.7 and those between +/-1.7 and +/-1.3 are depicted in this table. The regulated genes are sorted by their Identification number (ID). [file 1471-2199-11-40-S4.PDF]

Induced genes in Δ*lrpA1*

ID	log2	xfold	stdv	p-value	*gene*	protein name
OE1090F	0.6	1.5	0.3	6.24E-08	-	conserved hypothetical protein (nonfunctional
OE1156F	0.4	1.3	0.2	1.11E-08	*hsp20*	heat shock protein 20 homolog
OE1279R	0.5	1.4	0.3	9.22E-08	*rpoeps*	DNA-directed RNA polymerase (EC 2.7.7.6) epsilon subunit
OE1405R	0.7	1.6	0.2	4.04E-11	-	conserved hypothetical protein
OE1765R	0.5	1.4	0.1	4.66E-15	*pan2*	probable proteasome regulatory subunit
OE1781F	1.0	2.1	0.1	0	-	probable ABC-type transport system ATP-binding protein
OE1782F	0.7	1.6	0.2	9.70E-12	-	conserved hypothetical protein
OE1783F	0.9	1.8	0.1	0	-	conserved hypothetical protein
OE1794R	0.9	1.8	0.2	9.10E-15	-	conserved hypothetical protein
OE1797R	1.3	2.5	0.3	4.11E-14	*sirR*	transcription regulator sirR
OE2084R	0.5	1.4	0.2	7.58E-09	*tfbB*	transcription initiation factor TFB
OE2281R	0.5	1.4	0.2	2.13E-10	*tfbD*	transcription initiation factor TFB
OE2370R	0.5	1.4	0.2	8.45E-12	*gufA*	gufA protein
OE2618R	1.0	2.0	0.2	4.44E-16	-	conserved hypothetical protein
OE2619F	2.4	5.1	0.2	0	*aspB3*	probable aspartate aminotransferase (EC 2.6.1.1)
OE2886R	0.4	1.3	0.2	2.57E-07	-	probable transposase (ISH1)
OE2906R	0.4	1.4	0.4	3.74E-05	*sod2*	superoxide dismutase (EC 1.15.1.1) 2
OE3008F	0.4	1.4	0.3	6.46E-07	-	conserved hypothetical protein
OE3136F	1.2	2.4	0.3	4.24E-13	-	conserved hypothetical protein
OE3500R	0.4	1.4	0.1	7.09E-12	*nirH*	probable heme biosynthesis protein nirH/G
OE3659F	0.5	1.4	0.3	1.55E-07	-	conserved hypothetical protein
OE3815R	0.6	1.5	0.4	1.67E-06	-	conserved hypothetical protein
OE3901R	0.4	1.3	0.2	1.45E-08	*hat2*	probable N-acetyltransferase (EC 2.3.1.-)
OE4077F	0.5	1.4	0.2	3.00E-08	-	hypothetical protein
OE4189F	1.1	2.2	0.4	4.13E-11	-	conserved hypothetical protein
OE4196R	0.4	1.3	0.3	7.94E-07	-	conserved hypothetical protein
OE4217R	0.5	1.4	0.3	6.99E-07	*fdx*	ferredoxin (2Fe-2S)
OE4311F	0.7	1.7	0.2	2.88E-11	*appA*	ABC-type transport periplasmic substrate-binding protein
OE4427R	1.3	2.5	0.9	3.84E-06	*dpsA*	ferritin
OE4563F	0.6	1.5	0.3	1.70E-08	-	conserved hypothetical protein
OE4613F	0.9	1.9	0.5	4.53E-08	*acn*	aconitate hydratase (EC 4.2.1.3)
OE4622F	0.5	1.4	0.3	3.11E-06	-	conserved hypothetical protein
OE4648F	0.4	1.4	0.2	1.47E-07	*trxA2*	thioredoxin
OE4727R	0.8	1.7	0.4	8.77E-08	-	IS1341-type transposase (TCE31)
OE5048F	0.5	1.5	0.3	2.95E-08	-	conserved hypothetical protein
OE5083R	0.7	1.6	0.4	2.33E-06	*hsp5*	heat shock protein homolog
OE5162D1F	0.6	1.5	0.4	2.00E-06	-	conserved hypothetical protein
OE5186R	1.3	2.4	0.2	0	*perA*	catalase EC 1.11.1.6;including: peroxidase EC 1.11.1.7
OE5268R	0.4	1.4	0.1	1.14E-13	-	ABC-type transport system ATP-binding protein
OE6074R	0.6	1.5	0.1	9.01E-13	-	hypothetical protein
OE6093F	0.4	1.3	0.2	2.34E-08	*phzF*	probable phenazine biosynthesis protein
OE6130F	2.7	6.7	0.7	2.71E-13	-	conserved hypothetical protein
OE6156F	0.9	1.9	0.3	2.04E-11	-	conserved hypothetical protein
OE6166R	0.5	1.4	0.3	2.19E-06	-	conserved hypothetical protein (nonfunctional
OE7063A1R	0.4	1.4	0.3	2.17E-06	-	hypothetical protein
OE7068R	0.7	1.6	0.5	5.49E-06	-	conserved hypothetical protein
OE7190R	0.6	1.5	0.3	2.19E-08	-	hypothetical protein
OE7194F	0.5	1.4	0.2	2.17E-09	*repJ1*	plasmid replication protein repJ

Repressed genes in Δ*lrpA1*

ID	log2	fold	stdv	p-value	gene	protein name
OE1183F	-0.7	-1.6	0.1	6.00E-15	-	hypothetical protein

OE1294F	-0.6	-1.5	0.3	2.21E-09	-	ribosomal protein L15.eR
OE1919R	-0.5	-1.4	0.1	5.94E-13	-	conserved hypothetical protein
OE2385R	-0.5	-1.4	0.2	1.12E-10	*flaF*	fla operon protein flaF
OE2579F	-0.4	-1.3	0.1	9.40E-11	*purA*	adenylosuccinate synthase (EC 6.3.4.4)
OE2621R	-1.7	-3.2	0.6	1.52E-10	*lrpA1*	transcription regulator
OE3042F	-0.8	-1.7	0.7	4.04E-05	-	hypothetical protein
OE3106F	-0.8	-1.7	0.3	5.64E-11	*bop*	bacteriorhodopsin precursor
OE3116F	-0.6	-1.5	0.2	5.90E-11	-	conserved hypothetical protein
OE3277R	-0.4	-1.3	0.1	2.58E-14	*gcvH*	glycine cleavage system protein H
OE3318R	-0.6	-1.5	0.3	1.74E-08	*cbiN*	cobalt transport protein CbiN
OE3572R	-0.6	-1.5	0.2	9.52E-10	*pyrG*	CTP synthase (EC 6.3.4.2)
OE4114F	-0.4	-1.3	0.1	7.36E-13	*oxdhB*	2-oxoacid dehydrogenase (EC 1.2.4.-)
OE5071F	-1.3	-2.4	0.7	6.68E-08	-	protein kinase weak homolog
OE5204R	-0.6	-1.5	0.2	2.66E-12	*arcD*	arginine/ornithine antiporter
OE5448R	-0.6	-1.5	0.2	4.97E-11	-	IS1341-type transposase (nonfunctional, C-terminal part)
OE6308F	-1.0	-2.0	0.5	2.73E-08	-	hypothetical protein
OE7037F	-0.5	-1.4	0.2	1.39E-08	*gvpN1*	gas-vesicle operon protein gvpN
OE7038F	-0.6	-1.5	0.4	1.22E-05	*gvpO1*	gas-vesicle operon protein gvpO1
OE7039F	-1.3	-2.5	0.7	6.86E-08	*parA7*	parA domain protein
OE7042R	-1.2	-2.3	0.4	4.70E-11	-	signal-transducing histidine kinase/response regulator
OE7043A1F	-2.4	-5.4	0.4	2.22E-16	-	conserved hypothetical protein
OE7089R	-0.7	-1.7	0.3	6.17E-10	-	conserved hypothetical protein
OE7093R	-0.8	-1.8	0.2	1.62E-12	*idi1a*	isopentenyl-diphosphate delta-isomerase (EC 5.3.3.2) 1
OE7141R	-0.5	-1.4	0.2	1.63E-08	-	multidrug resistance transport protein homolog
OE7180F	-0.7	-1.6	0.3	3.37E-08	-	hypothetical protein
OE7212B1F	-0.9	-1.9	0.3	3.98E-12	-	conserved hypothetical protein
