# Supplementary material for: Transcriptional control by two leucine-responsive regulatory proteins in Halobacterium salinarum R1
Source: BMC Mol Biol. 2010 May 28;11:40. doi: 10.1186/1471-2199-11-40 (PMC2894021; doi:10.1186/1471-2199-11-40)
Supplement: Additional file 5 — RT-qPCR data compared to microarray data in ΔlrpA1 compared against wild type R1. Additional table S2 shows a comparison of RT-qPCR data with the microarray data. Total RNA was isolated from the deletion mutant ΔlrpA1 and wild type at a cell density OD600 0.4. We determined the transcript amount of the genes aspB3 and OE6130F, which encodes for a conserved hypothetical protein. [file 1471-2199-11-40-S5.PDF]

Additional Tab.S2 Comparison of RT-qPCR data with microarray data of the deletion mutant *ΔlrpA1* against wild type

ID	gene	name	fold change	fold change
***ΔlrpA1* /R1**			**RT-qPCR**	**microarray**
OE2619F	*aspB3*	aspartate transaminase	19	5.1
OE6130F	-	conserved hypothetical protein	28	6.7
