# Supplementary material for: Transcriptional control by two leucine-responsive regulatory proteins in Halobacterium salinarum R1
Source: BMC Mol Biol. 2010 May 28;11:40. doi: 10.1186/1471-2199-11-40 (PMC2894021; doi:10.1186/1471-2199-11-40)
Supplement: Additional file 9 — Differentially expressed genes in Δlrp. All significantly differentially expressed genes in Δlrp having a ratio higher than +/-1.7 and those between +/-1.7 and +/-1.3 are depicted in this table. The regulated genes are sorted by their Identification number (ID). [file 1471-2199-11-40-S9.PDF]

Induced genes in Δ*lrp*

ID	log2	xfold	stdv	p-value	gene	protein name
OE1014R	0.9	1.9	0.1	9.61E-14	*graD5*	glucose-1-phosphate thymidylyltransferase (EC 2.7.7.24)
OE1059R	1.3	2.5	0.2	3.42E-14	-	conserved hypothetical protein (nonfunctional
OE1060F	0.4	1.3	0.3	1.76E-05	-	hypothetical protein
OE1116F	0.5	1.4	0.2	1.32E-08	-	conserved hypothetical protein
OE1151R	0.5	1.4	0.2	1.30E-08	-	aminopeptidase homolog
OE1164R	1.0	2.0	0.3	1.71E-09	-	phosphatase homolog
OE1165R	1.0	2.0	0.2	1.06E-10	*serA3*	phosphoglycerate dehydrogenase (EC 1.1.1.95)
OE1188F	0.8	1.7	0.1	9.46E-13	-	conserved hypothetical protein
OE1205R	0.6	1.5	0.1	5.59E-12	-	conserved hypothetical protein
OE1211F	0.6	1.5	0.3	2.72E-06	*hsp4*	heat shock protein homolog
OE1254R	0.9	1.9	0.1	1.65E-13	-	conserved hypothetical protein
OE1259R	0.8	1.7	0.1	8.24E-14	-	conserved hypothetical protein
OE1260R	0.9	1.8	0.1	2.46E-12	*merA*	mercury(II) reductase homolog
OE1299R	0.5	1.4	0.1	1.28E-10	*hop*	halorhodopsin
OE1362R	0.7	1.6	0.3	1.68E-07	-	conserved hypothetical protein
OE1368R	0.7	1.7	0.1	1.57E-11	-	conserved hypothetical protein
OE1371R	0.7	1.6	0.1	7.58E-13	*pfdB*	prefoldin beta subunit
OE1405R	1.0	2.0	0.3	4.66E-10	-	conserved hypothetical protein
OE1410F	0.9	1.8	0.3	2.01E-08	-	conserved hypothetical protein
OE1414R	1.0	2.0	0.2	4.60E-12	*ftsZ4*	cell division protein ftsZ4
OE1425F	0.7	1.6	0.1	1.77E-12	-	conserved hypothetical protein
OE1435R	0.7	1.7	0.3	2.38E-08	-	conserved hypothetical protein
OE1461R	0.6	1.5	0.2	6.39E-09	-	conserved hypothetical protein
OE1480F	0.7	1.6	0.2	2.29E-08	-	ABC-type transport system substrate-binding protein
OE1490R	0.6	1.5	0.2	5.21E-08	-	conserved hypothetical protein
OE1510F	0.6	1.5	0.4	1.93E-05	-	conserved hypothetical protein
OE1520R	0.7	1.7	0.4	3.08E-06	-	hypothetical protein
OE1536R	0.8	1.7	0.4	1.20E-06	*htr14*	transducer protein htr14
OE1539F	1.3	2.5	0.1	2.22E-16	-	conserved hypothetical protein
OE1540R	0.9	1.8	0.3	1.33E-08	-	conserved hypothetical protein
OE1541F	0.6	1.5	0.2	3.71E-10	-	rhomboid family protein
OE1547R	0.4	1.4	0.2	6.34E-08	*arsA1*	probable anion-transporting ATPase (EC 3.6.1.-)
OE1550F	0.5	1.4	0.2	1.00E-07	-	conserved hypothetical protein
OE1566F	1.1	2.1	0.3	2.40E-09	-	hypothetical protein
OE1613R	0.5	1.5	0.2	4.07E-09	-	probable acylaminoacyl-peptidase (EC 3.4.19.1)
OE1632R	0.7	1.7	0.2	9.31E-10	*cyc*	unspecific monooxygenase (EC 1.14.14.1)
OE1641R	0.8	1.8	0.1	6.28E-14	*fad2*	3-hydroxybutryl-CoA dehydratase (EC 4.2.1.55)
OE1668R	0.4	1.4	0.1	2.53E-10	-	conserved hypothetical protein
OE1669F	0.4	1.4	0.2	2.87E-08	-	glucose 1-dehydrogenase homolog
OE1698R	1.4	2.7	0.3	1.25E-11	*oxr4*	probable oxidoreductase (EC 1.1.1.-)
OE1722R	1.0	2.0	0.2	1.00E-10	-	homolog to N-acetyltransferase
OE1726F	0.6	1.5	0.2	5.15E-09	*acs1*	acetate--CoA ligase (EC 6.2.1.1)
OE1748R	0.5	1.4	0.2	1.11E-08	*cna*	rRNA methylase homolog
OE1752F	0.5	1.4	0.2	2.73E-09	*ppd*	3-isopropylmalate dehydratase homolog
OE1761R	0.9	1.8	0.3	1.15E-07	-	conserved hypothetical protein
OE1763F	1.1	2.1	0.2	7.58E-13	-	conserved hypothetical protein
OE1765R	1.0	2.0	0.2	1.79E-11	*pan2*	probable proteasome regulatory subunit
OE1778R	0.9	1.8	0.2	1.46E-09	*oxr6*	probable oxidoreductase (EC 1.1.1.-)
OE1792F	0.9	1.9	0.4	6.62E-07	-	conserved hypothetical protein
OE1793F	0.6	1.5	0.2	1.08E-09	-	conserved hypothetical protein
OE1806R	1.1	2.1	0.2	5.17E-11	-	probable periplasmic protein
OE1823F	0.7	1.6	0.2	1.15E-08	-	hypothetical protein
OE1858F	0.6	1.5	0.1	4.35E-11	-	conserved hypothetical protein

OE1888R	0.4	1.4	0.2	4.17E-08	-	conserved hypothetical protein
OE1898A1F	0.6	1.5	0.1	1.06E-10	-	hypothetical protein
OE1899R	1.3	2.4	0.6	4.38E-07	-	conserved hypothetical protein
OE1900F	0.7	1.7	0.1	7.77E-13	-	conserved hypothetical protein
OE1903F	0.8	1.7	0.2	3.02E-10	-	glycine-rich protein
OE1934R	1.0	1.9	0.2	1.31E-11	*edp*	proteinase IV (EC 3.4.-.-)
OE1936R	1.1	2.1	0.2	7.31E-13	-	conserved hypothetical protein
OE1944R	0.5	1.4	0.1	5.71E-10	*aspB2*	probable aspartate aminotransferase (EC 2.6.1.1)
OE1947F	1.1	2.1	0.3	2.54E-09	-	conserved hypothetical protein
OE1969F	1.2	2.3	0.2	3.40E-12	-	CBS domain protein
OE1978R	0.6	1.5	0.1	6.88E-15	-	hypothetical protein
OE2006F	0.4	1.4	0.3	1.80E-05	-	probable ABC-type transport system ATP-binding protein
OE2007F	0.7	1.6	0.2	2.83E-10	-	probable hydrolase
OE2010R	0.5	1.4	0.1	3.84E-14	-	conserved hypothetical protein
OE2024F	0.6	1.5	0.3	1.55E-06	-	conserved hypothetical protein
OE2041R	0.6	1.5	0.1	1.38E-11	-	conserved hypothetical protein
OE2047R	0.6	1.5	0.2	1.00E-09	-	conserved hypothetical protein
OE2054F	0.7	1.6	0.2	5.63E-09	-	conserved hypothetical protein
OE2055F	0.6	1.5	0.1	5.33E-12	-	conserved hypothetical protein
OE2058R	0.8	1.8	0.1	7.64E-14	*afsQ2*	probable signal-transducing histidine kinase
OE2097F	0.5	1.4	0.2	6.34E-07	-	upsA domain protein
OE2118F	0.4	1.3	0.1	1.73E-09	*tfeA*	transcription initiation factor IIE alpha subunit homolog
OE2128F	0.9	1.9	0.2	1.82E-12	-	phoU protein homolog
OE2133R	0.5	1.4	0.2	3.19E-06	*aldH2*	aldehyde dehydrogenase (EC 1.2.1.-)
OE2170R	0.6	1.5	0.1	1.68E-10	*tmpC*	probable periplasmic substrate-binding protein
OE2186R	0.8	1.7	0.4	1.11E-06	*tatA*	sec-independent protein translocase component tatA
OE2187F	0.5	1.4	0.1	3.63E-11	-	conserved hypothetical protein
OE2199F	0.8	1.8	0.1	8.88E-16	*adh5*	probable alcohol dehydrogenase (EC 1.1.1.1)
OE2217R	0.6	1.6	0.4	3.55E-05	*mobA*	molybdopterin-guanine dinucleotide biosynthesis protein A
OE2225F	0.7	1.6	0.2	5.37E-09	*dmsB*	dimethylsulfoxide reductase (EC 1.8.-.-) subunit B
OE2243R	0.5	1.4	0.3	7.60E-06	*cynT*	probable carbonate dehydratase (EC 4.2.1.1)
OE2249R	0.6	1.5	0.2	3.59E-07	-	transducer protein weak homolog
OE2254R	0.6	1.5	0.3	4.37E-06	-	conserved hypothetical protein
OE2259F	0.5	1.4	0.3	6.30E-06	-	conserved hypothetical protein
OE2306F	0.5	1.4	0.3	2.93E-06	-	conserved hypothetical protein
OE2311R	0.5	1.4	0.2	2.26E-08	*udp2*	uridine phosphorylase (EC 2.4.2.3)
OE2319R	0.7	1.6	0.2	5.39E-10	-	conserved hypothetical protein
OE2332F	0.9	1.9	0.4	4.94E-07	*hakA*	probable kynureninase (EC 3.7.1.3)
OE2343R	0.4	1.4	0.1	1.41E-08	-	probable ABC-type transport system ATP-binding protein
OE2358F	0.5	1.4	0.1	3.61E-11	*acaB2*	probable nonspecific lipid-transfer protein
OE2367F	1.7	3.2	0.2	2.58E-14	*aldH3*	aldehyde dehydrogenase
OE2378R	0.4	1.3	0.1	1.53E-08	*parA1*	parA domain protein
OE2427F	0.6	1.5	0.1	1.39E-10	-	conserved hypothetical protein
OE2450F	1.3	2.5	0.3	9.25E-12	*acs2*	acetate-CoA ligase (EC 6.2.1.1)
OE2451R	0.9	1.8	0.2	5.90E-12	-	probable oxidoreductase
OE2453R	0.5	1.4	0.1	3.38E-11	-	conserved hypothetical protein
OE2455F	0.8	1.7	0.4	3.16E-06	-	conserved hypothetical protein
OE2513F	0.9	1.9	0.5	3.28E-06	-	conserved hypothetical protein
OE2514F	0.4	1.3	0.1	4.03E-11	-	hypothetical protein
OE2515F	1.0	2.0	0.4	3.54E-08	-	conserved hypothetical protein
OE2521R	0.5	1.4	0.2	2.83E-06	-	conserved hypothetical protein
OE2524R	0.9	1.9	0.2	4.37E-10	*udg1*	UDPglucose 6-dehydrogenase (EC 1.1.1.22)
OE2527F	1.0	2.0	0.3	7.79E-10	-	conserved hypothetical protein
OE2538R	0.4	1.3	0.2	1.55E-06	-	conserved hypothetical protein
OE2573F	0.4	1.3	0.3	3.87E-05	-	conserved hypothetical protein
OE2585R	0.8	1.8	0.4	1.39E-06	-	conserved hypothetical protein

OE2612F	0.5	1.4	0.1	6.35E-09	-	conserved hypothetical protein
OE2618R	0.8	1.7	0.1	8.32E-12	-	conserved hypothetical protein
OE2622R	0.5	1.4	0.2	8.62E-08	*porB*	pyruvate-ferredoxin oxidoreductase (EC 1.2.7.1) β-subunit
OE2623R	0.5	1.4	0.1	1.02E-09	*porA*	pyruvate-ferredoxin oxidoreductase (EC 1.2.7.1) α-subunit
OE2642R	0.4	1.4	0.1	4.11E-12	-	hypothetical protein
OE2648F	0.9	1.9	0.1	6.99E-14	-	conserved hypothetical protein
OE2674R	0.5	1.4	0.1	1.53E-09	*ksgA*	rRNA (adenine-N6
OE2676R	1.1	2.2	0.3	9.76E-11	-	conserved hypothetical protein
OE2695F	0.8	1.7	0.2	2.02E-10	*flgXXX*	flagellin flgXXX precursor
OE2696F	1.2	2.3	0.3	3.70E-11	-	conserved hypothetical protein
OE2697R	1.5	2.8	0.2	3.77E-15	*pqqE2*	probable coenzyme PQQ synthesis protein E
OE2698R	1.6	3.1	0.1	2.22E-16	-	hypothetical protein
OE2700F	1.8	3.6	0.1	0	*pqqE1*	probable coenzyme PQQ synthesis protein E
OE2703F	2.8	6.9	0.6	4.16E-11	-	probable copper-containing oxidoreductase
OE2708R	1.1	2.1	0.4	3.41E-08	*sod1*	superoxide dismutase (EC 1.15.1.1) 1 (Mn containing)
OE2710F	0.7	1.6	0.2	6.42E-09	*acd3*	probable acyl/butyryl-CoA dehydrogenase (EC 1.3.99.-)
OE2712R	0.4	1.3	0.2	1.90E-06	-	probable signal-transducing histidine kinase
OE2734F	0.6	1.5	0.1	7.34E-12	*hutU*	urocanate hydratase (EC 4.2.1.49)
OE2762R	0.7	1.6	0.2	9.36E-10	*aroA*	3-phosphoshikimate 1-carboxyvinyltransferase (EC 2.5.1.19)
OE2808F	1.3	2.5	0.5	1.09E-07	-	conserved hypothetical protein
OE2809R	0.7	1.7	0.3	1.93E-08	-	translation initiation factor aIF-2 beta subunit
OE2813R	0.4	1.3	0.2	4.94E-06	-	conserved hypothetical protein
OE2821F	1.3	2.4	0.2	9.60E-12	-	conserved hypothetical protein
OE2825F	0.4	1.4	0.1	1.93E-09	*moaC*	molybdenum cofactor biosynthesis protein C
OE2827R	1.1	2.2	0.2	3.34E-12	*hflX2*	probable GTP-binding protein
OE2828R	1.2	2.2	0.3	1.87E-09	-	conserved hypothetical protein
OE2856F	0.4	1.4	0.1	9.05E-09	-	conserved hypothetical protein
OE2863R	0.6	1.5	0.1	4.43E-11	-	conserved hypothetical protein
OE2872F	1.4	2.7	0.4	3.93E-09	-	conserved hypothetical protein
OE2874F	0.7	1.6	0.1	1.34E-11	-	conserved hypothetical protein
OE2900F	0.8	1.7	0.4	2.88E-06	-	conserved hypothetical protein
OE2906R	1.4	2.7	0.4	2.11E-09	*sod2*	superoxide dismutase (EC 1.15.1.1) 2
OE2909F	0.7	1.7	0.2	8.80E-10	-	conserved hypothetical protein
OE2922F	0.8	1.7	0.2	3.59E-09	-	conserved hypothetical protein
OE2961F	0.6	1.5	0.1	3.05E-11	*kinA1*	probable signal-transducing histidine kinase
OE2973F	1.8	3.5	0.3	6.11E-13	-	conserved hypothetical protein
OE2988R	0.6	1.5	0.1	1.01E-11	-	conserved hypothetical protein
OE2989R	1.1	2.1	0.2	3.04E-11	-	conserved hypothetical protein
OE2991F	1.2	2.3	0.2	2.60E-13	-	transcription factor TFB homolog
OE3008F	1.0	1.9	0.3	7.74E-09	-	conserved hypothetical protein
OE3030R	0.7	1.6	0.2	3.04E-10	-	conserved hypothetical protein
OE3065R	0.6	1.5	0.1	1.12E-10	*serA2*	phosphoglycerate dehydrogenase (EC 1.1.1.95)
OE3073R	1.8	3.4	0.4	3.52E-11	-	dodecin
OE3093R	1.0	2.0	0.3	6.22E-10	*crtB1*	phytoene synthase (EC 2.5.1.32)
OE3095R	1.5	2.9	0.3	1.68E-11	-	conserved hypothetical protein
OE3101R	1.0	2.0	0.4	3.43E-07	*bat*	bacterioopsin activator
OE3102R	0.8	1.8	0.3	7.86E-09	*brp*	bop gene expression regulator brp
OE3106F	1.6	2.9	0.2	8.88E-16	*bop*	bacteriorhodopsin precursor
OE3107F	0.5	1.4	0.1	2.21E-10	-	conserved hypothetical protein
OE3136F	3.8	13.9	0.6	6.17E-13	-	conserved hypothetical protein
OE3152R	0.5	1.4	0.1	1.96E-10	*pelA*	probable peptide chain release factor aRF1
OE3153R	0.5	1.4	0.1	2.59E-10	-	conserved hypothetical protein
OE3187R	0.9	1.9	0.3	7.53E-08	-	upsA domain protein
OE3203R	1.3	2.4	0.3	1.50E-10	-	conserved hypothetical protein
OE3204R	1.6	3.0	0.3	3.88E-12	-	conserved hypothetical protein
OE3207F	0.5	1.4	0.1	8.29E-12	*cbiT*	probable precorrin-8W decarboxylase (EC 1.-.-.-)

OE3229R	0.5	1.4	0.1	5.37E-12	*hmcA*	probable chelatase (EC 4.99.1.-) (cobalamin cluster)
OE3278R	0.5	1.4	0.1	1.48E-13	*gcvT*	glycine cleavage system protein T (EC 2.1.2.10)
OE3284R	0.9	1.9	0.1	8.54E-13	-	conserved hypothetical protein
OE3304R	0.6	1.5	0.2	3.10E-08	-	conserved hypothetical protein
OE3305F	0.6	1.5	0.1	1.51E-12	*rfcB*	replication factor C large subunit
OE3334R	0.9	1.9	0.1	0	*trpD1*	anthranilate phosphoribosyltransferase (EC 2.4.2.18)
OE3347F	0.8	1.8	0.2	1.11E-09	*htrI*	transducer protein htrI
OE3349F	1.2	2.3	0.2	1.67E-12	-	coenzyme F420-quinone oxidoreductase 42K s.u. homolog
OE3381R	0.4	1.4	0.2	8.13E-08	*crtI1*	phytoene dehydrogenase (EC 1.14.99.-)
OE3424R	0.9	1.9	0.3	8.00E-09	-	conserved hypothetical protein
OE3447F	0.6	1.6	0.1	2.06E-12	-	conserved hypothetical protein
OE3451A1F	0.7	1.6	0.1	1.16E-12	-	conserved hypothetical protein
OE3458R	0.5	1.4	0.1	2.77E-10	-	probable oxidoreductase (versicolorin reductase homolog)
OE3462R	0.7	1.6	0.2	3.12E-09	-	hypothetical protein
OE3491R	0.7	1.6	0.1	1.32E-11	-	heat shock protein homolog
OE3500R	0.4	1.3	0.1	1.24E-08	*nirH*	probable heme biosynthesis protein nirH/G
OE3513R	0.7	1.7	0.3	9.88E-08	-	hypothetical protein
OE3541R	1.7	3.3	0.3	1.86E-12	*hsp1*	probable heat shock protein
OE3542R	1.9	3.8	0.6	1.94E-09	-	glutamine-rich alkaline protein
OE3562R	0.5	1.4	0.1	1.49E-09	-	conserved hypothetical protein
OE3565F	0.8	1.8	0.2	2.91E-11	*moaB*	molybdenum cofactor biosynthesis protein B
OE3566F	0.4	1.3	0.1	1.67E-11	-	conserved hypothetical protein
OE3595R	0.6	1.5	0.2	3.57E-10	*moaD*	molybdopterin (MPT) converting factor
OE3612R	0.8	1.8	0.1	3.39E-13	*basB*	chemotactic signal transduction protein basB
OE3659F	0.4	1.4	0.1	7.95E-11	-	conserved hypothetical protein
OE3661F	0.4	1.3	0.1	2.28E-10	*trp2*	ABC-type transport system ATP-binding protein
OE3668F	1.5	2.8	0.2	1.32E-12	-	upsA domain protein
OE3681R	0.6	1.5	0.3	4.40E-07	-	conserved hypothetical protein
OE3691F	0.9	1.8	0.4	5.07E-07	*trpD2*	anthranilate phosphoribosyltransferase (EC 2.4.2.18)
OE3714F	1.4	2.6	0.1	2.22E-16	-	conserved hypothetical protein
OE3717F	0.9	1.8	0.1	2.44E-15	*nolA*	NADH dehydrogenase (ubiquinone) (EC 1.6.5.3) 32K subunit
OE3718F	0.7	1.6	0.1	5.11E-15	*ftsZ3*	cell division protein
OE3728R	0.8	1.7	0.2	1.44E-09	-	conserved hypothetical protein
OE3752R	0.7	1.6	0.2	1.94E-10	-	mutT domain protein
OE3766R	0.8	1.7	0.2	2.57E-09	-	hypothetical protein
OE3798R	0.6	1.5	0.3	3.08E-06	-	conserved hypothetical protein
OE3807R	0.4	1.3	0.2	4.96E-07	*yjbG*	oligoendopeptidase
OE3814R	0.6	1.5	0.4	2.31E-05	-	conserved hypothetical protein
OE3815R	1.8	3.4	0.2	2.66E-15	-	conserved hypothetical protein
OE3836F	0.8	1.7	0.2	5.66E-10	*yyaI*	probable acetyltransferase
OE3843F	0.8	1.7	0.2	1.28E-09	*nadE*	NAD+ synthase (EC 6.3.1.5)
OE3846R	0.5	1.4	0.1	1.87E-11	*fad1*	enoyl/3-hydroxybutryl-CoA dehydratase (EC 4.2.1.-)
OE3864R	1.0	2.0	0.3	3.71E-10	-	conserved hypothetical protein
OE3892R	0.4	1.4	0.1	2.02E-09	-	beta-lactamase homolog
OE3903F	0.8	1.8	0.5	2.00E-05	-	conserved hypothetical protein
OE3925R	0.4	1.4	0.3	2.35E-05	*cctB*	thermosome beta subunit
OE3942R	2.2	4.5	0.3	3.89E-14	-	hypothetical protein
OE3946F	1.0	1.9	0.2	1.66E-10	-	conserved hypothetical protein
OE3947R	0.5	1.4	0.1	3.98E-11	-	conserved hypothetical protein
OE3998R	0.5	1.4	0.1	4.66E-10	-	conserved hypothetical protein
OE4011R	0.5	1.4	0.2	1.61E-07	-	conserved hypothetical protein
OE4018F	0.5	1.4	0.2	5.28E-07	-	conserved hypothetical protein
OE4032R	0.6	1.5	0.2	4.94E-09	-	conserved hypothetical protein
OE4033R	0.7	1.6	0.1	1.01E-13	*dbp*	probable DNA helicase (enhancer-binding protein homolog)
OE4039F	0.6	1.5	0.1	1.13E-14	-	conserved hypothetical protein
OE4052F	0.5	1.4	0.1	4.66E-10	*mcm*	DNA helicase (EC 3.6.1.-) mcm (intein-containing)

OE4056R	0.4	1.3	0.1	1.77E-10	*tfbA*	transcription initiation factor TFB
OE4065R	0.8	1.7	0.3	1.86E-07	*hit1*	histidine triad family protein
OE4073R	0.9	1.8	0.3	8.41E-08	*hcpB*	halocyanin hcpB
OE4077F	1.9	3.9	0.2	5.77E-15	-	hypothetical protein
OE4080F	0.5	1.4	0.3	1.69E-05	-	conserved hypothetical protein
OE4099R	0.5	1.4	0.2	4.60E-08	-	conserved hypothetical protein
OE4113F	0.5	1.4	0.2	2.72E-08	*oxdhA1*	2-oxoacid dehydrogenase (EC 1.2.4.-)
OE4122R	0.9	1.9	0.3	4.21E-09	*cctA*	thermosome alpha subunit
OE4127R	0.5	1.4	0.1	4.44E-12	-	conserved hypothetical protein
OE4146F	1.2	2.3	0.2	1.26E-11	*tbpE*	TATA-binding transcription initiation factor
OE4152R	0.4	1.3	0.1	4.54E-10	*hisG*	ATP phosphoribosyltransferase (EC 2.4.2.17)
OE4159F	1.3	2.5	0.1	0	*achY*	adenosylhomocysteinase (EC 3.3.1.1)
OE4184F	0.8	1.8	0.1	6.45E-13	*orc6*	cell division control protein cdc6 homolog
OE4190F	0.9	1.8	0.2	3.65E-12	*pmm*	probable phosphohexomutase (EC 5.4.2.-)
OE4196R	1.0	1.9	0.3	1.21E-09	-	conserved hypothetical protein
OE4218F	1.2	2.2	0.2	3.10E-13	*hisA*	PRFPR-imidazolecarboxamide isomerase (EC 5.3.1.16)
OE4240F	0.6	1.5	0.1	1.55E-10	-	conserved hypothetical protein
OE4244F	0.4	1.3	0.1	5.95E-11	-	conserved hypothetical protein
OE4276F	0.4	1.4	0.1	1.65E-12	*hemC*	hydroxymethylbilane synthase (EC 4.3.1.8)
OE4283R	0.8	1.7	0.2	4.72E-10	-	signal-transducing histidine kinase / response regulator
OE4296F	0.6	1.5	0.3	4.37E-06	-	hypothetical protein
OE4320R	1.0	2.0	0.4	2.93E-08	-	conserved hypothetical protein
OE4325F	0.7	1.6	0.2	7.87E-10	*rad24b*	probable DNA helicase
OE4331R	0.5	1.4	0.1	2.89E-09	*tnaA*	tryptophanase (EC 4.1.99.1)
OE4365F	0.7	1.6	0.2	2.45E-08	-	conserved hypothetical protein
OE4384R	1.6	3.0	0.4	6.59E-11	-	conserved hypothetical protein
OE4399F	0.5	1.5	0.1	9.10E-15	*oxr8*	probable oxidoreductase (EC 1.1.1.-)
OE4414R	0.7	1.6	0.3	3.04E-07	-	salinity-regulated protein homolog
OE4418R	0.4	1.3	0.1	1.46E-10	-	hypothetical protein
OE4419R	0.4	1.4	0.1	1.73E-08	*argH*	argininosuccinate lyase (EC 4.3.2.1)
OE4420R	1.2	2.3	0.2	2.05E-11	*argG*	argininosuccinate synthase (EC 6.3.4.5)
OE4436R	0.4	1.3	0.1	1.39E-10	*lta*	L-allo-threonine aldolase (EC 4.2.1.-)
OE4449R	0.5	1.4	0.1	3.51E-12	-	hypothetical protein
OE4456R	0.5	1.4	0.2	8.73E-08	*gimC*	prefoldin alpha subunit
OE4459R	0.6	1.5	0.1	1.80E-10	*rpl31R*	ribosomal protein L31.eR
OE4461F	0.5	1.4	0.2	7.48E-09	-	conserved hypothetical protein
OE4492F	0.4	1.4	0.1	2.45E-12	-	conserved hypothetical protein
OE4496R	0.6	1.5	0.2	8.52E-08	*fer4*	ferredoxin (3Fe-4S)(4Fe-4S)
OE4500R	0.6	1.6	0.2	1.79E-09	*acd6*	probable acyl/butyryl-CoA dehydrogenase (EC 1.3.99.-)
OE4509F	1.6	3.0	0.5	2.06E-08	-	nonhistone chromosomal protein
OE4529F	0.4	1.3	0.2	3.29E-06	*aldH1*	aldehyde dehydrogenase (EC 1.2.1.-)
OE4543R	0.5	1.5	0.2	1.06E-07	*rimI1*	probable N-terminal acetyltransferase
OE4544R	0.8	1.7	0.1	3.79E-13	-	upsA domain protein
OE4562R	0.7	1.6	0.1	4.54E-13	*entB*	N-carbamoylsarcosine amidase homolog
OE4563F	0.5	1.4	0.3	2.82E-05	-	conserved hypothetical protein
OE4565F	0.6	1.5	0.3	1.55E-07	-	conserved hypothetical protein
OE4612F	1.2	2.2	0.2	3.63E-13	*hly*	halolysin R4 (EC 3.4.21.-)
OE4621F	0.6	1.5	0.3	2.65E-07	-	ADP-ribose pyrophosphatase homolog
OE4633F	0.6	1.5	0.1	1.15E-10	-	conserved hypothetical protein
OE4637R	0.6	1.5	0.3	8.04E-06	*gbp4*	probable GTP-binding protein
OE4638F	0.6	1.5	0.2	3.04E-07	-	hypothetical protein
OE4648F	0.6	1.5	0.2	6.62E-10	*trxA2*	thioredoxin
OE4650R	0.8	1.7	0.3	2.45E-08	-	hypothetical protein
OE4651F	0.4	1.3	0.2	2.26E-07	-	probable ribose-1
OE4654F	0.4	1.3	0.1	6.71E-10	*thiD*	phosphomethylpyrimidine kinase (EC 2.7.4.7)
OE4665R	0.4	1.4	0.1	2.40E-14	*hflX1*	GTP-binding protein

OE4671R	0.4	1.4	0.2	6.00E-08	-	conserved hypothetical protein
OE4707R	1.4	2.7	0.2	5.68E-13	-	conserved hypothetical protein
OE4759F	0.5	1.5	0.4	4.49E-05	*csg*	cell surface glycoprotein precursor
OE5106F	0.5	1.4	0.3	7.82E-06	*trkA7*	trkA domain protein
OE5136R	0.9	1.9	0.2	1.58E-11	-	conserved hypothetical protein
OE5142F	0.4	1.3	0.1	1.48E-10	*ftsZ5*	cell division protein ftsZ5
OE5160F	1.3	2.5	0.1	0	*gldA1*	glycerol dehydrogenase (EC 1.1.1.6)
OE5162D1F	2.5	5.7	0.6	2.72E-10	-	conserved hypothetical protein
OE5178F	0.5	1.4	0.1	9.27E-09	-	conserved hypothetical protein
OE5186R	0.4	1.4	0.1	3.13E-13	*perA*	catalase (EC 1.11.1.6) (including: peroxidase (EC 1.11.1.7))
OE5187R	0.7	1.6	0.1	3.44E-11	-	probable hydrolase
OE5204R	1.0	2.0	0.2	1.09E-12	*arcD*	arginine/ornithine antiporter
OE5205R	1.2	2.3	0.2	1.86E-12	*arcB*	ornithine carbamoyltransferase (EC 2.1.3.3)
OE5243F	0.8	1.7	0.2	6.58E-10	*car*	transducer protein car
OE5276F	0.4	1.3	0.2	2.53E-07	-	conserved hypothetical protein
OE5322R	0.5	1.4	0.2	1.19E-06	-	conserved hypothetical protein
OE5378R	0.5	1.4	0.2	7.15E-07	-	hypothetical protein
OE6308F	1.6	3.0	0.2	2.02E-14	-	hypothetical protein
OE6345R	0.6	1.5	0.3	6.66E-06	-	hypothetical protein
OE6352F	1.8	3.5	0.5	7.66E-10	-	IS1341-type transposase (TCE32)
OE7065F	0.7	1.7	0.2	1.18E-10	*cydA1*	cytochrome d ubiquinol oxidase (EC 1.10.3.-) subunit I
OE7129F	0.5	1.4	0.3	2.13E-05	-	conserved hypothetical protein
OE7187F	0.9	1.9	0.4	2.03E-07	-	conserved hypothetical protein

Repressed genes in Δ*lrp*

ID	log2	fold	stdv	p-value	gene	protein name
OE1004F	-0.4	-1.4	0.1	1.36E-10	-	probable ABC-type transport system ATP-binding protein
OE1018F	-0.7	-1.6	0.1	2.15E-11	-	sugar transferase
OE1031F	-1.4	-2.6	0.4	5.31E-10	-	probable transposase (ISH10)
OE1047R	-0.7	-1.7	0.2	7.61E-09	-	hypothetical protein
OE1054F	-0.7	-1.6	0.3	3.00E-07	-	hypothetical protein
OE1055R	-0.5	-1.4	0.1	5.66E-12	-	conserved hypothetical protein
OE1076F	-1.0	-2.0	0.2	3.30E-11	-	cell division control protein cdc6 homolog
OE1078F	-0.5	-1.4	0.2	1.03E-07	*graD6*	glucose-1-phosphate thymidylyltransferase (EC 2.7.7.24)
OE1080F	-0.6	-1.5	0.3	4.08E-06	-	probable polysaccharide export protein
OE1094R	-1.9	-3.7	0.2	7.11E-15	-	probable transposase (ISH10)
OE1100F	-0.7	-1.7	0.2	3.74E-09	-	hypothetical protein
OE1101R	-0.4	-1.4	0.2	2.20E-07	-	hypothetical protein
OE1107R	-0.4	-1.4	0.2	1.69E-07	-	conserved hypothetical protein
OE1112R	-0.6	-1.5	0.2	2.57E-08	*lpb*	probable glycosyltransferase (EC 2.-.-.-)
OE1121F	-1.3	-2.5	0.4	6.66E-09	-	conserved hypothetical protein
OE1134F	-0.5	-1.4	0.2	1.51E-08	-	conserved hypothetical protein
OE1162R	-0.7	-1.6	0.3	1.88E-06	*cspD1*	probable cold shock protein
OE1171F	-0.6	-1.5	0.1	6.25E-12	*rmeMa*	type I restriction-modification system DNA-methyltransferase
OE1183F	-0.5	-1.4	0.1	1.82E-12	-	hypothetical protein
OE1197R	-0.6	-1.5	0.2	5.66E-08	-	hypothetical protein
OE1226F	-0.5	-1.4	0.2	6.14E-07	-	conserved hypothetical protein
OE1239F	-1.2	-2.3	0.1	4.44E-16	-	hypothetical protein
OE1294R	-0.7	-1.6	0.2	3.37E-10	*rpl15R*	ribosomal protein L15.eR
OE1372R	-0.6	-1.5	0.2	1.24E-09	*rpoP*	DNA-directed RNA polymerase (EC 2.7.7.6) subunit P
OE1439F	-0.7	-1.7	0.3	1.25E-07	-	probable IS200-type transposase (TCE31)
OE1462R	-0.5	-1.4	0.2	1.44E-07	-	nicotinamide-nucleotide adenylyltransferase (EC 2.7.7.1)
OE1478R	-0.6	-1.6	0.2	1.95E-09	*tfbF*	transcription initiation factor TFB
OE1505F	-0.8	-1.8	0.1	2.26E-12	-	probable 5'-methylthioadenosine phosphorylase (nonfunctional)
OE1506R	-0.5	-1.4	0.2	2.11E-07	-	probable transposase (ISH8/ISH26)

OE1553F	-0.6	-1.5	0.2	1.95E-07	-	conserved hypothetical protein
OE1560R	-0.4	-1.4	0.1	6.84E-09	-	conserved hypothetical protein
OE1584R	-1.3	-2.5	0.2	3.45E-13	-	conserved hypothetical protein
OE1620R	-0.9	-1.8	0.1	5.94E-13	*purH*	p-ribosylglycinamide formyltransferase (EC 2.1.2.2)
OE1623F	-1.0	-2.0	0.2	4.26E-11	*purB*	adenylosuccinate lyase (EC 4.3.2.2)
OE1675R	-0.5	-1.4	0.2	1.59E-08	*pstB2*	ABC-type phosphate transport system ATP-binding protein
OE1678R	-0.9	-1.8	0.2	2.02E-09	*pstC2*	probable ABC-type phosphate transport system permease protein
OE1684F	-0.9	-1.9	0.2	2.00E-12	*nodP*	probable sulfate adenylyltransferase (EC 2.7.7.4) small subunit
OE1687F	-0.8	-1.8	0.1	1.55E-13	*aspS*	aspartate-tRNA ligase (EC 6.1.1.12)
OE1707R	-0.5	-1.5	0.1	3.51E-10	-	transcription regulator homolog
OE1710R	-0.9	-1.9	0.2	1.58E-11	*korB*	oxoglutarate-ferredoxin oxidoreductase (EC 1.2.7.3) β-subunit
OE1711R	-1.5	-2.9	0.4	5.56E-10	*korA*	oxoglutarate-ferredoxin oxidoreductase (EC 1.2.7.3) α-subunit
OE1797R	-0.8	-1.8	0.2	8.68E-10	*sirR*	transcription regulator sirR
OE1817R	-0.6	-1.5	0.2	8.60E-09	-	conserved hypothetical protein
OE1840R	-0.4	-1.3	0.2	2.81E-08	*apt*	purine phosphoribosyltransferase (EC 2.4.2.7)
OE1867R	-0.6	-1.5	0.3	3.01E-06	-	conserved hypothetical protein
OE1872R	-0.5	-1.4	0.1	9.13E-11	*petD*	probable menaquinol-cytochrome-c reductase (EC 1.10.2.-)
OE1919R	-0.6	-1.5	0.1	1.97E-13	-	conserved hypothetical protein
OE1951F	-0.8	-1.7	0.2	1.75E-10	*purK*	phosphoribosylaminoimidazole carboxylase (EC 4.1.1.21)
OE1956F	-0.4	-1.4	0.2	3.39E-07	*nuoCD*	NADH dehydrogenase (ubiquinone) (EC 1.6.5.3) subunit CD
OE1959F	-0.7	-1.7	0.4	5.02E-06	*nuoJ1*	NADH dehydrogenase (ubiquinone) (EC 1.6.5.3) subunit J1
OE1964F	-0.6	-1.5	0.4	1.71E-05	*nuoL*	NADH dehydrogenase (ubiquinone) (EC 1.6.5.3) subunit L
OE1981R	-0.6	-1.5	0.1	5.68E-11	-	hypothetical protein
OE2020F	-0.5	-1.5	0.1	1.28E-11	*fbp*	fructose-bisphosphatase (EC 3.1.3.11)
OE2044F	-0.6	-1.5	0.2	4.60E-09	-	probable heavy metal binding protein
OE2046F	-0.8	-1.8	0.2	2.15E-10	-	conserved hypothetical protein
OE2130F	-0.6	-1.5	0.1	6.49E-11	-	conserved hypothetical protein
OE2142R	-0.6	-1.5	0.2	3.57E-08	-	phospholipase D
OE2165R	-0.7	-1.7	0.1	1.17E-12	*rps15*	ribosomal protein S15
OE2230F	-0.8	-1.7	0.1	3.50E-13	-	conserved hypothetical protein
OE2267F	-0.4	-1.3	0.2	2.61E-08	*rpoL*	DNA-directed RNA polymerase (EC 2.7.7.6) subunit L
OE2268R	-0.4	-1.3	0.2	7.43E-07	*hisF*	imidazoleglycerol-phosphate synthase (EC 2.4.2.-) subunit hisF
OE2269F	-0.6	-1.5	0.1	6.06E-14	-	hypothetical protein
OE2274R	-1.0	-2.0	0.1	2.48E-13	*purL*	phosphoribosylformylglycinamidine synthase (EC 6.3.5.3)
OE2307F	-0.7	-1.6	0.2	2.25E-08	-	NADH dehydrogenase homolog
OE2309F	-0.5	-1.5	0.2	3.76E-08	-	hypothetical protein
OE2310F	-0.5	-1.4	0.1	2.81E-10	-	conserved hypothetical protein
OE2348R	-0.8	-1.7	0.1	6.09E-13	-	probable ABC-type transport system substrate-binding protein
OE2374R	-0.5	-1.4	0.1	3.81E-10	*cheW2*	purine-binding chemotaxis protein cheW2
OE2406R	-1.0	-2.0	0.1	0	*cheR*	protein-glutamate O-methyltransferase (EC 2.1.1.80) cheR
OE2432C1F	-0.8	-1.7	0.3	1.12E-07	-	hypothetical protein
OE2458R	-0.5	-1.4	0.2	9.16E-09	*guaB*	IMP dehydrogenase (EC 1.1.1.205)
OE2460F	-0.5	-1.4	0.1	4.04E-13	-	conserved hypothetical protein
OE2465R	-0.5	-1.4	0.1	2.09E-12	-	hypothetical protein
OE2473F	-0.5	-1.4	0.3	1.08E-05	-	glutaredoxin homolog
OE2530F	-0.5	-1.5	0.1	1.74E-11	*graD4*	glucose-1-phosphate thymidylyltransferase (EC 2.7.7.24)
OE2531F	-0.8	-1.7	0.1	1.59E-13	-	conserved hypothetical protein
OE2533F	-1.7	-3.3	0.1	0	-	hypothetical protein
OE2536F	-0.4	-1.3	0.2	2.31E-07	-	hypothetical protein
OE2537F	-0.5	-1.4	0.1	5.66E-09	*rfbQ*	sugar transferase homolog
OE2540R	-0.5	-1.5	0.2	4.38E-08	-	probable transposase (ISH6/ISHS1)
OE2547R	-0.6	-1.5	0.2	2.50E-09	*exoM*	probable glycosyltransferase (EC 2.-.-.-)
OE2569R	-0.4	-1.3	0.2	7.60E-08	-	sulfite oxidase homolog
OE2572F	-0.7	-1.6	0.2	1.90E-09	-	conserved hypothetical protein
OE2579F	-0.5	-1.5	0.1	1.16E-09	*purA*	adenylosuccinate synthase (EC 6.3.4.4)
OE2603R	-0.7	-1.6	0.2	1.33E-08	*rpl11*	ribosomal protein L11

OE2605R	-0.6	-1.5	0.1	2.28E-10	-	conserved hypothetical protein
OE2627F	-0.7	-1.6	0.2	5.19E-09	*rps13*	ribosomal protein S13
OE2629F	-0.9	-1.8	0.5	3.54E-06	*rps11*	ribosomal protein S11
OE2631F	-1.2	-2.3	0.1	4.44E-16	*rpoD*	DNA-directed RNA polymerase (EC 2.7.7.6) subunit D
OE2632F	-1.3	-2.5	0.1	2.22E-16	*rpl18R*	ribosomal protein L18.eR
OE2633F	-1.2	-2.4	0.3	3.75E-11	*rpl13*	ribosomal protein L13
OE2635F	-0.8	-1.8	0.4	2.98E-06	*rps9*	ribosomal protein S9
OE2637F	-1.7	-3.2	0.2	4.44E-16	*rpoN*	DNA-directed RNA polymerase (EC 2.7.7.6) subunit N
OE2640F	-0.7	-1.7	0.3	4.10E-08	*eno*	enolase (EC 4.2.1.11)
OE2641F	-1.4	-2.6	0.2	1.09E-14	*rps2*	ribosomal protein S2
OE2683R	-0.7	-1.6	0.2	2.54E-08	*aef1b*	translation elongation factor aEF-1 beta subunit
OE2715R	-0.5	-1.4	0.1	3.19E-10	-	hypothetical protein
OE2716R	-0.4	-1.3	0.3	2.33E-05	-	hypothetical protein
OE2727R	-0.5	-1.4	0.2	9.45E-07	-	conserved hypothetical protein
OE2740F	-1.0	-2.0	0.3	1.59E-09	-	conserved hypothetical protein
OE2744R	-0.4	-1.3	0.2	1.23E-06	-	conserved hypothetical protein
OE2745R	-0.5	-1.4	0.3	5.24E-06	*pgk*	phosphoglycerate kinase (EC 2.7.2.3)
OE2750R	-0.6	-1.5	0.2	4.18E-10	-	conserved hypothetical protein
OE2753F	-0.8	-1.8	0.1	2.37E-12	*orc8*	cell division control protein cdc6 homolog
OE2779F	-0.6	-1.6	0.3	2.02E-06	*pheP*	amino acid transport protein (probable phenylalanine)
OE2780F	-0.8	-1.8	0.2	2.81E-09	-	hypothetical protein
OE2782F	-1.1	-2.1	0.3	7.28E-09	*surE*	probable stationary-phase survival protein
OE2847R	-0.8	-1.7	0.2	8.91E-10	-	conserved hypothetical protein
OE2853R	-0.6	-1.5	0.3	6.04E-06	*slyD*	probable peptidylprolyl isomerase (EC 5.2.1.8)
OE2865R	-0.6	-1.5	0.3	1.59E-06	*sdhA*	succinate dehydrogenase (EC 1.3.99.1) subunit A (flavoprotein)
OE2903R	-0.5	-1.4	0.2	9.18E-09	-	conserved hypothetical protein
OE2946R	-0.6	-1.5	0.3	6.03E-07	-	conserved hypothetical protein
OE3017R	-1.1	-2.2	0.1	5.33E-15	*ush*	UDP-sugar hydrolase (EC 3.6.1.45) / 5'-nucleotidase (EC 3.1.3.5)
OE3062F	-0.5	-1.4	0.3	8.96E-06	*rps17R*	ribosomal protein S17.eR
OE3063F	-0.5	-1.4	0.1	8.32E-11	*asd*	aspartate-semialdehyde dehydrogenase (EC 1.2.1.11)
OE3069R	-0.7	-1.6	0.2	1.53E-09	-	conserved hypothetical protein
OE3116F	-0.9	-1.8	0.3	2.76E-09	-	conserved hypothetical protein
OE3118F	-0.6	-1.5	0.3	7.97E-07	-	conserved hypothetical protein
OE3132F	-0.6	-1.5	0.2	8.05E-08	-	conserved hypothetical protein
OE3139R	-1.6	-2.9	0.3	7.69E-12	*purF*	amidophosphoribosyltransferase (EC 2.4.2.14)
OE3141R	-1.1	-2.2	0.1	1.13E-14	*rpl37R*	ribosomal protein L37.eR
OE3142R	-0.9	-1.9	0.2	1.53E-11	*snp*	SM protein
OE3155R	-0.8	-1.8	0.2	6.81E-10	-	conserved hypothetical protein
OE3262R	-0.9	-1.9	0.1	7.25E-13	-	conserved hypothetical protein
OE3317R	-0.6	-1.5	0.1	7.15E-10	*cbiQ*	probable cobalt transport protein CbiQ
OE3319R	-0.5	-1.4	0.1	1.53E-10	*cbiM*	CbiM protein (unassigned function)
OE3337F	-0.8	-1.8	0.2	1.37E-11	-	insertion element protein (ISH2)
OE3384R	-0.6	-1.5	0.2	4.13E-08	-	conserved hypothetical protein
OE3390F	-0.8	-1.7	0.2	3.01E-11	*rpl23*	ribosomal protein L23
OE3394F	-0.8	-1.8	0.2	4.22E-11	*rpl22*	ribosomal protein L22
OE3395F	-0.5	-1.4	0.1	3.37E-10	*rps3*	ribosomal protein S3
OE3396F	-0.5	-1.4	0.2	8.14E-08	*rpl29*	ribosomal protein L29
OE3404F	-0.5	-1.4	0.2	3.44E-07	*rpl24*	ribosomal protein L24
OE3405F	-0.6	-1.5	0.3	2.18E-06	*rps4R*	ribosomal protein S4.eR
OE3407F	-0.5	-1.4	0.2	7.10E-07	*rpl5*	ribosomal protein L5
OE3429F	-0.6	-1.5	0.3	9.32E-07	*cmk*	cytidylate kinase (EC 2.7.4.14)
OE3481R	-0.6	-1.5	0.2	5.07E-08	*htrII*	transducer protein htrII
OE3486R	-0.5	-1.4	0.2	6.35E-08	*speB*	agmatinase (EC 3.5.3.11)
OE3487R	-0.6	-1.5	0.3	1.31E-06	*eif5a*	translation initiation factor aIF-5A
OE3547F	-0.6	-1.5	0.3	3.47E-06	-	conserved hypothetical protein
OE3554F	-0.9	-1.9	0.5	1.92E-06	*carB*	carbamoyl-phosphate synthase (EC 6.3.-.-) large subunit

OE3556R	-1.5	-2.8	0.1	1.11E-15	*carA*	carbamoyl-phosphate synthase (EC 6.3.-.-) small subunit
OE3558F	-0.7	-1.6	0.1	1.64E-12	*trh3*	probable transcription regulator
OE3560F	-0.5	-1.4	0.2	3.99E-08	*idi3*	probable isopentenyl-diphosphate delta-isomerase (EC 5.3.3.2) 3
OE3571R	-0.5	-1.4	0.2	2.76E-08	*guaAb*	GMP synthase (glutamine-hydrolyzing) (EC 6.3.5.2)
OE3572R	-1.4	-2.6	0.3	4.69E-11	*pyrG*	CTP synthase (EC 6.3.4.2)
OE3582F	-0.7	-1.7	0.3	5.43E-07	*cspD2*	probable cold shock protein
OE3639F	-0.4	-1.4	0.3	1.12E-05	-	conserved hypothetical protein
OE3652F	-0.5	-1.4	0.1	5.99E-13	-	small multidrug export protein
OE3654R	-0.8	-1.8	0.1	6.37E-14	-	hypothetical protein
OE3655R	-0.4	-1.3	0.1	3.01E-08	-	hypothetical protein
OE3688F	-0.4	-1.3	0.1	9.02E-10	-	conserved hypothetical protein
OE3712R	-0.6	-1.5	0.2	1.98E-09	*oxdhA2*	2-oxoacid dehydrogenase (EC 1.2.4.-)
OE3724F	-0.8	-1.7	0.4	4.16E-06	*purC*	p-ribosylaminoimidazolesuccinocarboxamide synthase (EC 6.3.2.6)
OE3731R	-1.6	-2.9	0.3	3.02E-11	*purQ*	phosphoribosylformylglycinamidine synthase (EC 6.3.5.3)
OE3732R	-1.3	-2.4	0.3	1.04E-09	-	conserved hypothetical protein
OE3749R	-0.5	-1.4	0.3	3.30E-05	*tgtA1*	queuine tRNA-ribosyltransferase (EC 2.4.2.29)
OE3784R	-0.5	-1.5	0.2	2.20E-09	*secD*	preprotein-export translocase subunit secD
OE3787R	-0.5	-1.4	0.3	1.94E-05	*secF*	preprotein-export translocase subunit secF
OE3822R	-0.7	-1.7	0.3	1.71E-07	-	conserved hypothetical protein
OE3829R	-0.4	-1.4	0.1	3.92E-12	-	conserved hypothetical protein
OE3866R	-0.4	-1.4	0.1	1.78E-14	*gcp*	probable O-sialoglycoprotein endopeptidase (EC 3.4.24.57)
OE3907R	-0.8	-1.7	0.1	1.83E-12	*phnE*	ABC-type transport system permease protein
OE3908R	-0.8	-1.8	0.2	1.44E-10	*phnC*	ABC-type transport system ATP-binding protein
OE3922R	-0.6	-1.5	0.3	1.85E-05	*glnA*	glutamate--ammonia ligase (EC 6.3.1.2)
OE3923F	-3.1	-8.8	1.1	3.99E-08	*lrp*	global transcription regulator
OE3950R	-1.9	-3.8	0.4	4.62E-11	-	conserved hypothetical protein
OE3963R	-2.6	-6.1	0.4	7.05E-13	*ribB*	3,4-dihydroxy-2-butanone 4-phosphate synthase (EC 5.4.99.-)
OE3964R	-2.9	-7.5	0.2	0	-	conserved hypothetical protein
OE3984R	-0.5	-1.4	0.1	1.11E-10	*atpB*	H+-transporting two-sector ATPase (EC 3.6.3.14) subunit B.a
OE3985R	-0.6	-1.6	0.2	1.62E-09	*atpA*	H+-transporting two-sector ATPase (EC 3.6.3.14) subunit A.a
OE3986R	-0.9	-1.9	0.1	5.57E-13	*atpF*	H+-transporting two-sector ATPase (EC 3.6.3.14) subunit F.a
OE3987R	-0.4	-1.4	0.2	2.31E-06	*atpC*	H+-transporting two-sector ATPase (EC 3.6.3.14) subunit C.a
OE4012F	-0.4	-1.3	0.1	1.14E-08	-	conserved hypothetical protein
OE4047R	-0.6	-1.5	0.1	4.72E-12	-	phage PhiH1 repressor protein homolog
OE4049R	-0.5	-1.5	0.2	2.45E-09	-	hypothetical protein
OE4187R	-0.7	-1.6	0.4	9.90E-06	-	probable DNA-binding protein
OE4201R	-0.5	-1.4	0.2	1.48E-06	*flaK*	probable preflagellin peptidase
OE4223R	-0.6	-1.5	0.2	1.18E-08	-	conserved hypothetical protein
OE4300R	-0.9	-1.8	0.1	3.75E-14	-	conserved hypothetical protein
OE4301R	-1.1	-2.2	0.1	2.22E-16	*ykfD*	ABC-type transport system ATP-binding protein
OE4302R	-1.3	-2.4	0.1	8.88E-16	*oppD2*	ABC-type oligopeptide transport system ATP-binding protein
OE4303R	-0.9	-1.9	0.2	5.07E-12	*dppC2*	ABC-type transport system permease protein
OE4304R	-0.9	-1.9	0.3	1.43E-08	*dppB1*	ABC-type transport system permease protein
OE4367R	-0.4	-1.3	0.1	2.28E-08	-	conserved hypothetical protein
OE4380F	-0.6	-1.5	0.2	1.09E-08	*orc7*	cell division control protein cdc6 homolog
OE4385F	-0.5	-1.4	0.1	5.98E-11	-	conserved hypothetical protein
OE4387F	-0.5	-1.4	0.3	1.00E-05	-	hypothetical protein
OE4435F	-0.9	-1.9	0.2	2.23E-10	-	hypothetical protein
OE4523F	-0.4	-1.3	0.3	4.95E-05	-	hypothetical protein
OE4524F	-0.5	-1.4	0.1	3.81E-09	-	hypothetical protein
OE4552F	-1.2	-2.3	0.4	1.97E-08	*dppB2*	ABC-type transport system permease protein
OE4572R	-0.9	-1.9	0.1	2.51E-13	*valS*	valine-tRNA ligase (EC 6.1.1.9)
OE4585R	-0.4	-1.3	0.2	3.80E-07	-	hypothetical protein
OE4610R	-0.7	-1.6	0.1	9.10E-15	-	dCTP deaminase homolog
OE4628R	-0.7	-1.6	0.2	2.79E-10	-	conserved hypothetical protein

OE4683F	-2.9	-7.6	0.1	0	*ribC*	riboflavin synthase (EC 2.5.1.9) alpha subunit
OE4720R	-1.0	-2.0	0.2	1.63E-10	*rps10*	ribosomal protein S10
OE4721R	-0.8	-1.7	0.2	1.50E-10	*aef1a*	translation elongation factor aEF-1 alpha subunit
OE4735R	-0.6	-1.5	0.2	1.82E-09	*rps7*	ribosomal protein S7
OE4740R	-0.5	-1.4	0.1	1.48E-12	*rpoA1*	DNA-directed RNA polymerase (EC 2.7.7.6) subunit A' (chain A)
OE4741R	-0.5	-1.4	0.3	3.70E-06	*rpoB1*	DNA-directed RNA polymerase (EC 2.7.7.6) subunit B'
OE5015F	-0.7	-1.6	0.3	3.92E-08	-	probable transposase (ISH8/ISH26)
OE5022F	-0.8	-1.7	0.3	2.90E-08	-	conserved hypothetical protein
OE5031R	-0.8	-1.8	0.1	1.57E-12	-	conserved hypothetical protein
OE5062R	-0.6	-1.5	0.2	1.05E-08	-	IS1341-type transposase (TCE31)
OE5065R	-0.6	-1.5	0.1	3.80E-11	-	conserved hypothetical protein
OE5143F	-0.5	-1.4	0.3	9.41E-06	-	probable transport protein
OE5162R	-0.7	-1.6	0.1	1.91E-14	*orc5*	cell division control protein cdc6 homolog
OE5201F	-0.9	-1.9	0.2	1.35E-10	*pyrB*	aspartate carbamoyltransferase (EC 2.1.3.2) catalytic subunit
OE5202F	-1.0	-2.0	0.3	1.50E-08	*pyrI*	aspartate carbamoyltransferase (EC 2.1.3.2) regulatory subunit
OE5211F	-0.7	-1.6	0.1	7.23E-13	-	conserved hypothetical protein
OE5234R	-0.6	-1.5	0.2	3.13E-07	-	conserved hypothetical protein (nonfunctional
OE5260F	-1.2	-2.3	0.2	2.49E-14	-	probable transposase (ISH10)
OE5268R	-0.5	-1.4	0.1	7.43E-11	-	ABC-type transport system ATP-binding protein
OE5279R	-1.1	-2.2	0.3	1.14E-09	-	conserved hypothetical protein
OE5307F	-0.4	-1.3	0.1	1.58E-11	-	ABC-type transport system ATP-binding protein
OE5325F	-0.4	-1.4	0.2	3.13E-07	-	conserved hypothetical protein
OE5338R	-1.5	-2.8	0.1	1.11E-15	-	transposase homolog (ISH8/ISH26) (nonfunctional
OE5340R	-0.6	-1.5	0.3	9.37E-07	-	transposase homolog (ISH8/ISH26) (nonfunctional
OE5363R	-0.9	-1.9	0.2	5.96E-11	-	insertion element protein (ISH2)
OE5370R	-1.1	-2.2	0.3	7.41E-10	-	hypothetical protein
OE5393F	-1.1	-2.2	0.2	3.70E-11	-	insertion element protein (ISH2)
OE5400F	-0.6	-1.5	0.2	1.12E-08	-	conserved hypothetical protein
OE5407F	-0.6	-1.5	0.1	4.77E-11	-	probable transposase (ISH8/ISH26)
OE5448F	-1.0	-1.9	0.2	2.48E-10	-	IS1341-type transposase (TCE32)
OE6005R	-0.5	-1.4	0.2	7.45E-08	-	conserved hypothetical protein
OE6006R	-0.7	-1.7	0.1	3.64E-14	-	conserved hypothetical protein
OE6012F	-1.1	-2.2	0.3	9.99E-11	-	probable transposase (ISH6/ISHS1)
OE6027R	-0.5	-1.4	0.1	3.08E-13	-	hypothetical protein
OE6032F	-0.4	-1.3	0.1	1.52E-09	-	conserved hypothetical protein
OE6034F	-0.8	-1.7	0.2	2.76E-10	-	IS1341-type transposase (TCE32)
OE6046F	-0.9	-1.9	0.2	2.02E-11	-	conserved hypothetical protein
OE6052R	-0.5	-1.4	0.3	8.81E-06	-	hypothetical protein
OE6074R	-1.5	-2.9	0.1	0	-	hypothetical protein
OE6075R	-0.6	-1.5	0.3	4.95E-06	-	probable transposase (ISH8/ISH26)
OE6079F	-0.5	-1.4	0.1	3.60E-09	-	conserved hypothetical protein
OE6096A1R	-0.8	-1.7	0.4	8.06E-07	-	conserved hypothetical protein
OE6099F	-0.7	-1.6	0.1	1.71E-12	-	hypothetical protein
OE6111A1F	-0.6	-1.5	0.0	0	-	conserved hypothetical protein
OE6113F	-0.9	-1.8	0.3	5.91E-09	-	conserved hypothetical protein
OE6128R	-0.4	-1.3	0.1	2.00E-09	-	conserved hypothetical protein
OE6130F	-1.8	-3.5	0.2	2.55E-14	-	conserved hypothetical protein
OE6133R	-1.2	-2.3	0.4	5.03E-09	-	probable transposase (ISH10)
OE6138F	-0.4	-1.4	0.1	6.50E-09	-	conserved hypothetical protein
OE6145R	-1.0	-2.0	0.2	1.49E-12	-	probable signal-transducing histidine kinase
OE6154F	-0.7	-1.6	0.1	7.11E-12	-	hypothetical protein
OE6156F	-1.1	-2.1	0.3	1.25E-09	-	conserved hypothetical protein
OE6161R	-0.7	-1.6	0.3	9.38E-08	-	conserved hypothetical protein (nonfunctional
OE6166R	-0.8	-1.7	0.2	4.22E-10	-	conserved hypothetical protein (nonfunctional
OE6276R	-0.8	-1.8	0.2	2.89E-09	-	transposase homolog (TCE33) (nonfunctional)
OE6283R	-0.8	-1.7	0.2	8.56E-09	-	IS200-type transposase homolog (TCE31) (nonfunctional

OE6285F	-0.6	-1.5	0.2	1.08E-09	-	IS1341-type transposase (TCE31)
OE6292R	-0.8	-1.7	0.3	9.45E-08	-	probable transposase (ISH6/ISHS1)
OE6296R	-0.6	-1.5	0.2	1.37E-07	-	probable transposase (ISH8/ISH26)
OE6298A1R	-1.5	-2.7	0.2	6.41E-13	-	insertion element protein (ISH2)
OE7001R	-0.4	-1.4	0.2	7.24E-07	-	conserved hypothetical protein
OE7008F	-0.7	-1.6	0.2	2.22E-10	-	hypothetical protein (encoded by ISH7/ISH24 subtype 1)
OE7011R	-0.4	-1.3	0.1	2.10E-08	-	hypothetical protein
OE7015F	-0.5	-1.4	0.3	4.51E-05	-	conserved hypothetical protein (nonfunctional
OE7023R	-0.7	-1.6	0.3	4.84E-07	*gvpL1*	gas-vesicle operon protein gvpL1
OE7024R	-0.7	-1.7	0.2	3.02E-11	*gvpK1*	gas-vesicle operon protein gvpK1
OE7026R	-0.6	-1.6	0.2	4.97E-09	*gvpJ1*	gas-vesicle operon protein gvpJ1
OE7027R	-0.5	-1.4	0.3	3.79E-05	*gvpI1*	gas-vesicle operon protein gvpI1
OE7034F	-2.0	-4.0	0.7	4.02E-08	*gvpA1*	gas-vesicle protein gvpA1
OE7034F	-1.0	-2.0	0.3	6.10E-09	*gvpA1*	gas-vesicle protein gvpA1
OE7036F	-1.5	-2.8	0.5	5.29E-09	*gvpC1*	gas-vesicle protein gvpC1
OE7037F	-1.5	-2.8	0.2	6.44E-15	*gvpN1*	gas-vesicle operon protein gvpN
OE7038F	-1.6	-3.0	0.5	1.70E-08	*gvpO1*	gas-vesicle operon protein gvpO1
OE7039F	-3.5	-11.6	0.3	0	*parA7*	parA domain protein
OE7042R	-3.2	-9.1	0.8	6.08E-10	-	probable signal-transducing histidine kinase / response regulator
OE7043A1F	-3.1	-8.3	0.4	2.11E-14	-	conserved hypothetical protein
OE7045F	-0.5	-1.5	0.1	5.85E-10	*tbpA*	TATA-binding transcription initiation factor homolog
OE7049R	-0.6	-1.5	0.4	1.60E-05	-	conserved hypothetical protein (nonfunctional
OE7052F	-0.5	-1.4	0.2	5.87E-07	-	probable transposase (ISH5)
OE7054R	-0.4	-1.4	0.3	1.36E-05	-	conserved hypothetical protein
OE7056A1R	-0.8	-1.8	0.3	2.38E-08	-	conserved hypothetical protein
OE7057F	-0.8	-1.7	0.2	1.32E-10	-	conserved hypothetical protein
OE7064R	-0.5	-1.4	0.2	5.62E-07	-	hypothetical protein
OE7089R	-0.7	-1.7	0.1	1.69E-14	-	conserved hypothetical protein
OE7092F	-0.6	-1.6	0.2	1.96E-09	-	hypothetical protein
OE7095F	-0.4	-1.3	0.1	9.70E-14	-	conserved hypothetical protein
OE7097F	-1.8	-3.5	0.1	0	-	probable transposase (ISH10)
OE7100R	-0.7	-1.7	0.2	1.68E-10	*parA6a*	parA domain protein
OE7101R	-1.3	-2.5	0.6	5.01E-07	-	conserved hypothetical protein
OE7102R	-0.8	-1.8	0.1	9.33E-15	-	conserved hypothetical protein
OE7104R	-0.5	-1.4	0.3	1.56E-05	-	conserved hypothetical protein
OE7106F	-0.4	-1.4	0.1	6.41E-09	-	hypothetical protein
OE7116B1R	-0.4	-1.3	0.1	1.82E-08	-	conserved hypothetical protein
OE7141R	-1.0	-2.0	0.2	2.86E-12	-	multidrug resistance transport protein homolog
OE7144R	-0.5	-1.5	0.2	5.06E-08	-	conserved hypothetical protein
OE7149F	-0.6	-1.5	0.2	6.30E-09	-	hypothetical protein
OE7174R	-0.7	-1.6	0.2	2.80E-08	-	conserved hypothetical protein
OE7180F	-0.8	-1.7	0.2	5.51E-09	-	hypothetical protein
OE7182F	-1.1	-2.2	0.2	2.73E-11	-	probable restriction/modification enzyme
OE7186F	-1.1	-2.1	0.2	9.25E-13	*tbpC1*	probable TATA-binding transcription initiation factor
OE7189F	-0.4	-1.3	0.2	4.20E-06	-	conserved hypothetical protein
OE7190R	-0.9	-1.9	0.3	7.62E-09	-	hypothetical protein
OE7192F	-0.5	-1.4	0.1	1.24E-09	-	conserved hypothetical protein
OE7196F	-0.5	-1.4	0.2	4.58E-07	-	phage integrase homolog
OE7198F	-0.9	-1.8	0.3	4.41E-08	-	probable transposase (ISH8/ISH26)
OE7201R	-0.5	-1.4	0.1	5.85E-10	-	probable transposase (ISH4/ISH23/ISH50)
OE7210R	-0.5	-1.4	0.3	1.33E-06	-	conserved hypothetical protein
OE7212B1F	-1.0	-2.0	0.1	1.40E-14	-	conserved hypothetical protein
OE7215F	-1.2	-2.4	0.2	9.65E-12	-	conserved hypothetical protein
OE8005F	-0.4	-1.3	0.2	1.07E-07	-	conserved hypothetical protein
OE8009R	-0.6	-1.5	0.3	8.12E-07	-	conserved hypothetical protein
OE8047F	-1.3	-2.5	0.3	8.84E-11	-	conserved hypothetical protein

OE8050F	-0.6	-1.5	0.2	4.18E-08	-	conserved hypothetical protein
