# Supplementary material for: Transcriptional control by two leucine-responsive regulatory proteins in Halobacterium salinarum R1
Source: BMC Mol Biol. 2010 May 28;11:40. doi: 10.1186/1471-2199-11-40 (PMC2894021; doi:10.1186/1471-2199-11-40)
Supplement: Additional file 10 — A table of oligonucleotides. Oligonucleotides used in these experiments [file 1471-2199-11-40-S10.PDF]

Oligonucleotides used in this study (Sequence 5´⟶3´)*

Expressions of LrpA1 into the Pet26b vector

ExpLrpA1	fwd TTTTTTCATATGAGCACTGAATCGACAGAAGA
	rev TTTTTTCTCGAGCTTGAGGCGTTCCTGGAGGA

Northern blot: Probe against aspartate transaminase (OE2619F)

NbOE2619F	fwd ATGAGGGATGGCCGCGATCC
	rev TAATACGACTCACTATAGGGAGAGTCGGTGCGGTCGGCGAGG
NbOE2621R	fwd TCACTTGAGGCGTTCCTGGAG
	rev TAATACGACTCACTATAGGGAGAGTGAGCACTGAATCGACAGAAG

Construction of the deletion mutants

ΔOE2621R	fwd upstream TTTTTT**GGATCC**AGACATCGCCGACGAGCC
	rev upstream TTTTTT**CTGCAG**TTCGCTCTTCTGTCGATTCAGT
	fwd donwstream TTTTTT**CTGCAG**CCAGGAACGCCTCAAGTGAG PstI
	rev downstream TTTTTT**TCTAGA**GATCGGCGATGACACCGCC
ΔOE3923F	fwd upstream TTTTTT**GGATCC**TTCGGTGCGAGATCGAAG
	rev upstream TTTTTT**CTGCAG**AAGTTTACGAACGTCCAATTCTG
	fwd downstream TTTTTT**CTGCAG**CCGGGAACGCGACCGGACAC
	rev downstream TTTTTT**TCTAGA**CCGACCACGTCGTCGACG

probes for Southern blotting

ΔlrpA1	fwd CGTTTTGATTGTCGGCTACGTG
	rev CGGTGCGAACGCAACCGAGT
lrpA1	fwd TTTTTTCATATGAGCACTGAATCGACAGAAGA
	rev TTTTTTCTCGAGCTTGAGGCGTTCCTGGAGGA
Δlrp	fwd CTGCATGGATAAATGAGTATCGC
	rev ACGTTCTTTGCGCGCGATCTC
lrp	fwd ATGACCTACGAGAACCTCGACG
	rev CGCGTCGCCGTCGAGGTCGA

Construction of the overexpression mutants

↑*lrp*	Lrp_Pst_for GTAGCC**CTGCAG**ATGACCTACGAGAACCTC
	Lrp_Bam_rev TCGAAC**GGATCC**TTTTCGGCGGCCGCGTTG
↑*lrpA1*	LrpA1_Pst_for AATAT**CTGCAG**GTGAGCACTGAATCGACAG
	LrpA1_Bam_rev GCGTT**GGATCC**GGAACGAGGTCACCGAGCG
P*bop*_for:	AGTGGAAGCTTGCGTGCACGCATCGACTT
P*bop*_rev:	AACTCCTGCAGGCAACAGTACCTAACGAGGA
Lrp_rev:	ATCTCCACTGCACCAGCGGTGCCGC
LrpA1_rev:	ATTTCGGTGCGAACGCAACCGAGTA

DNA-binding-assay

Pro_lrpA1	fwd ACGACACCGGGTCGTTTTGAT
Pro_lrpA1bio	rev TGGAGACGTCCGCACGCTC
Promut_lrpA1	fwd AAGATACCCTTTCACACGAACC
Promut_lrpA1	rev GGATAGGGGGTTCGTGTGAAA
Pro_aspB3	fwd ATCGCGCTTGAACGTCTGCAT
Pro_aspB3bio	rev GTCGGCGGCGCGTTCGAG
flaA	fwd TCGGCACGCTCATCGTGTTCATCG

flaA2bio	rev GCGCTCGTCTTCGTCAGTGATGAACTCG

Reverse transcription-quantitative PCR

OE6130F	fwd GACGTGAGCGAAAATGGAGAC
	rev ACATTCCCGTCAACACCCTC
aspB3	fwd CTGTTGGCCACTGTCGACC
	rev AAGTTCGGGTAGCAGGCGTA
lrpA1	fwd CGACCGTCCGCAAATACAT
	rev TTTGCCCGGACAGCTTCTT
lrp	fwd CCTGAAATCACTGGGACGCT
	rev TCGGTGTCGGTGAACTTCC

5´-3´-RACE

a.) cDNA-Synthese

P1cDNALrpA1-(I)	GACGATGACGCCGTCCGATTCG
P2cDNALrpA1-(II)	CTTTGCCCGGACAGCTTCTTGG
P1cDNAasp-(I)	GGCTGGGGGTGCGAAGTCGG
P2cDNAasp-(II)	CGCGTCGACTGCTGCCTCGG

b.) 1. PCR und 2. „nested"-PCR

PCRLrpA1:	fwd CACATGCTGATGGCGGAGGT
	rev TCGATGTATTTGCGGACGGTC
NESPCRLrpA1:	fwd CTCGGTGACGTGATCAGCGAC
	rev ACGCTCCGCGATGGCCGCAT
PCRasp:	fwd CGGATTTCGGTGCGAACGCA
	rev CCACTTCCATGTGGATCACGT
NESPCRasp	fwdCGGGCGTCGTTTGCGGTGAG
	rev GTTCGAGGACATCCATCGCGG

* Bold sequences indicate restriction sites.
